# High *TLR7* Expression Drives the Expansion of CD19^+^CD24^hi^CD38^hi^ Transitional B Cells and Autoantibody Production in SLE Patients

**DOI:** 10.3389/fimmu.2019.01243

**Published:** 2019-06-04

**Authors:** Ting Wang, John Marken, Janice Chen, Van Bao Tran, Quan-Zhen Li, Mengtao Li, Karen Cerosaletti, Keith B. Elkon, Xiaofeng Zeng, Natalia V. Giltiay

**Affiliations:** ^1^Department of Rheumatology, Peking Union Medical College Hospital, Peking Union Medical College and Chinese Academy of Medical Sciences, Key Laboratory of Rheumatology and Clinical Immunology, Ministry of Education, Beijing, China; ^2^Division of Rheumatology, Department of Medicine, University of Washington, Seattle, WA, United States; ^3^Translational Research Program, Benaroya Research Institute at Virginia Mason, Seattle, WA, United States; ^4^Department of Immunology, University of Texas Southwestern Medical Center, Dallas, TX, United States

**Keywords:** TLR7, transitional B cells, SLE, autoantibodies, RNP, type I IFNs

## Abstract

Signaling through Toll-like receptor 7 (TLR7) drives the production of type I IFN and promotes the activation of autoreactive B cells and is implicated in the pathogenesis of systemic lupus erythematosus (SLE). While TLR7 has been extensively studied in murine lupus, much less is known about its role in the pathogenesis of human SLE. Genetic studies support a link between the *TLR7 rs3853839 C/G* polymorphism, which affects *TLR7* mRNA turnover, and SLE susceptibility; however, the effects of this polymorphism on B cells have not been studied. Here we determined how changes in *TLR7* expression affect peripheral B cells and auto-Ab production in SLE patients. High *TLR7* expression in SLE patients driven by *TLR7 rs3853839 C/G* polymorphism was associated with more active disease and upregulation of IFN-responsive genes. TLR7^hi^ SLE patients showed an increase in peripheral B cells. Most notably, the percentage and numbers of CD19^+^CD24^++^CD38^++^ newly-formed transitional (TR) B cells were increased in TLR7^hi^ SLE patients as compared to HCs and TLR7^norm/lo^ SLE patients. Using auto-Ab arrays, we found an increase and enrichment of auto-Ab specificities in the TLR7^hi^ SLE group, including the production of anti-RNA/RNP-Abs. Upon *in vitro* TLR7 ligand stimulation, TR B cells isolated from TLR7^hi^ but not TLR7^norm/lo^ SLE patients produced anti-nuclear auto-Abs (ANA). Exposure of TR B cells isolated from cord blood to IFNα induced the expression of *TLR7* and enabled their activation in response to TLR7 ligation *in vitro*. Our study shows that overexpression of *TLR7* in SLE patients drives the expansion of TR B cells. High TLR7 signaling in TR B cells promotes auto-Ab production, supporting a possible pathogenic role of TR B cells in human SLE.

## Introduction

Systemic lupus erythematosus (SLE) is a chronic autoimmune disease characterized by B cell hyperactivation, associated with the production of antinuclear autoantibodies (auto-Abs) and the formation of immune complexes (IC). A subset of SLE patients has auto-Abs, which are reactive to RNA and ribonucleoproteins (RNPs), including anti-Ro, anti-Sm, and anti-RNP and, are associated with increased expression of IFN-stimulated genes (ISGs) and worse disease severity (1–3). Despite their contribution to SLE pathogenesis, the immune sensors that drive the production of anti-RNA/RNP auto-Abs in human SLE are not well-established.

Toll-like receptor 7 (TLR7), an endosomal Toll-like receptor, specialized in the recognition of single-stranded RNA (ssRNA) appears to play a key role in SLE pathogenesis (4–8).

TLR7 mediates type I IFN production in human pDC (9, 10). Among their other functions, type I IFNs can activate B cells through receptor for IFN I (IFNAR) to induce upregulation of ISGs, including IFNAR and TLR7. Signaling *via* IFNAR affects BCR signaling, B cell selection and class-switch recombination (11). Increased type I IFNs can also, indirectly, promote B cell survival and activation by driving the production of B cell-activating factor (BAFF) and other cytokines by myeloid cells and/or T cells (12, 13).

Data from mouse lupus models support a B cell-intrinsic role of TLR7 signaling in B cell activation and the production of auto-Abs (7, 14–18). Increase in *Tlr7* gene dosage in non-autoimmune mice promotes the development of a lupus-like disease, whereas, the deletion of the *Tlr7* allele in lupus-prone mice eliminates anti-RNA auto-Abs and reduces disease pathology (4, 5, 7, 8). *Tlr7.1* transgenic (*Tlr7.1*Tg) mice, which express 8-16 extra copies of *Tlr7*, develop an early onset systemic autoimmune disease, associated with the production of anti-RNA auto-Abs. *Tlr7.1*Tg mice show expansion and activation of newly-formed transitional (TR) B cells, follicular B cell expansion, and increased germinal center (GC) formation (5, 15, 18).

Genetic studies in human SLE support a link between copy number variations (CNV) and single-gene polymorphisms (SNP) in the *TLR7* gene locus and SLE susceptibility (19–23). The *TLR7 rs3853839 C/G* SNP, located in the 3′ untranslated region of the *TLR7*, has been associated with an increase in *TLR7* mRNA and TLR7 protein expression and, upregulation of ISGs (22, 24).

While the role of TLR7 in B cells has been extensively investigated in murine lupus, still much less is known about the TLR7 signaling in human SLE. In this study, we explored how changes in *TLR7* expression, including an increase in *TLR7* due to *TLR7 rs3853839 C/G* polymorphism, affects peripheral B cells and auto-Ab production in SLE patients.

## Materials and Methods

### Study Subjects

SLE (*n* = 65) patients were recruited from the University of Washington Medical Center. All patients fulfilled the revised ACR criteria for SLE; disease activity was measured using the SLE Disease Activity Index (SLEDAI) 2K (25, 26). The mean SLEDAI of the study cohort was 5.2 ± 3.46 (range 1–16). Patients treated with biologics within the last 6 months or taking more than 40 mg prednisone per day or suspected of having acute infections were excluded from the analysis. Healthy controls (HC) (*n* = 16) with no history of autoimmune diseases or current infections were selected to match the ethnicity, age, and sex of the SLE patients. 47 SLE subjects were analyzed for the expression *TLR7* in PBMC and genotyped for the *TLR7* rs3853839 C/G polymorphism. Peripheral B cell phenotypes were analyzed in 12 HCs and 40 SLE subjects. Additional information about the study subjects, included in the analysis presented here is shown in Table 1, Supplemental Tables 1 and 2.

**Table 1:** Characteristics of the SLE patients according to the expression of *TLR7* in peripheral blood mononuclear cells.

**Category**	**Feature**	**TLR7 ^**norm/low**^** **(*n* = 21)**	**TLR7^**high**^** **(*n* = 19)**	***p*-value**
Demographic	Age, mean ± SD	40.50 ± 4.572	35.72 ± 3.072	NS
	No. (%) female	18 (85.71)	17 (89.47)	NS
	No. (%) males	3 (14.28)	2 (10.52)	NS
	Race/Ethnicity, No (%)			
	Asian	1 (4.8)	5 (26.31)	
	Black or African American	4 (19.05)	0 (0)	
	Caucasian	6 (28.57)	7 (36.84)	
	Hispanic	4 (19.05)	4 (21.05)	
	Native Hawaiian/PI	1 (4.8)	1 (5.2)	
	Unknown/other	5	2	
Disease	SLEDAI, mean ± SD	3.8 ± 2.5	7.2 ± 3.37	0.0189
	Current renal involvement, No (%)	1 (4.8)	6 (31.58)	0.0395
	Anti-dsDNA positive, No (%)	13 (61.9)	16 (84.21)	NS
	Lymphocyte, count ± SD	1.337 ± 0.2547	1.092 ± 0.1483	NS
Medications	HCQ, No (%)	15 (71.42)	16 (84.21)	NS
	Prednisone, currently taking, No. (%)	6 (28.57)	8 (42.10)	NS
	Prednisone, dose [mg ± SD]	5.364 ± 2.738	8.750 ± 3.000	NS
	Mycophenolate mofetil, or Cyclophosphamide exposure, No. (%)	0 (0)	6 (31.58)	0.0247

### Cell Isolation and Cell Culture

Whole blood from SLE patients and HCs was collected into sodium-heparinized tubes (BD Biosciences, San Jose, CA) and peripheral blood mononuclear cells (PBMCs) were purified by Ficoll-Hypaque (GE Healthcare Life Sciences, Marlborough, MA) density-gradient centrifugation. PBMCs were suspended in PBS, 2% FBS and analyzed by flow cytometry. 1–2 × 10^6^ cells from each sample were pelleted and stored at −80°C in RNA lysis buffer for RNA isolation. Umbilical cord blood (CB) was obtained from Bloodworks Northwest, Seattle. CD19^+^ CD38^++^ CD24^++^ TR B cells were purified from PBMC or CB by cell sorting performed on a FACSAria II high-speed sorter (BD Biosciences). B cell numbers in SLE patients were determined by multiplying clinical lymphocyte counts by the fraction CD19^+^ B cells of PBMCs, determined by flow cytometry. Upon sorting, cells were cultured with RPMI 1640 containing L-glutamine and NaHCO_3_, supplemented with 10% FCS (Atlanta Biologicals), and 1% penicillin/streptomycin (Sigma). PBMC TR B cells were stimulated with 50 ng/ml TLR7/8 agonist R848 (Resiquimod) (InvivoGen, San Diego, CA) in 96-well plates using 3–5 × 10^3^ cells/well in 100 μl medium and cultured for 5 days. Sort-purified CB TR B cells were cultured at 0.2–1 × 10^6^ in 200 μl medium, stimulated with IFNα at 1000 U/ml (PBL, Piscataway, NJ) for 6 h, washed, and then, stimulated with 50 ng/ml R848 alone, or R848 plus F(ab′)2 anti-human IgM (anti-IgM) (10 μg/ml) (Jackson ImmunoResearch Laboratories, Inc). For the cell proliferation experiments, cells were loaded with 2.5 μM CFSE (Thermo Fisher Scientific) in PBS at 37°C for 10 min, quenched with medium, washed, primed with IFNα then, washed and stimulated with R848, cultured for 3 days and analyzed for CFSE dilution by flow cytometry. IgM and IgG production by CB-TR B cells was measured using Ready-SET-Go® ELISA kits (e-Biosciences, Thermo Fisher Scientific).

### Flow Cytometry

Freshly isolated PBMCs were stained with combinations of fluorescently labeled mAbs. Live cells were identified using fixable viability dye (FVD) (Invitrogen, Thermo Fisher Scientific). Analysis of B cell subsets was performed using combinations of fluorescently-conjugated Abs (Supplemental Table 3). For the analysis of *TLR7* expression, cells were first stained with FVD, washed, stained with anti-CD19 and then, fixed, permeabilized and stained with PE-conjugated anti-TLR7 or PE-isotype control antibodies using transcription factor buffer set (BD Biosciences). Sm/RNP (Arotec Diagnostics, Wellington, New Zealand) was labeled with Alexa Fluor® 647 Antibody Labeling Kit (Molecular Probes, Eugene, OR) and used in combination with appropriate surface markers for detection of Sm/RNP^+^ CD19^+^ B cells. Flow cytometry was performed using a 5-laser LSRII flow cytometer (BD) or a 4-laser CytoFLEX flow cytometer (Beckman Coulter, Brea, CA). All data were analyzed using FlowJo software (Tree Star, Ashland, OR).

### Real-Time PCR

Total RNA was extracted from PBMCs or sorted B cells using RNA Purification Plus Kit (Norgen Biotek, Thorold, ON). Reverse transcription reactions were prepared using 100 ng of total RNA per reaction using High-Capacity cDNA Reverse Transcription Kit (Applied Biosystems, Foster City, CA). Gene expression was analyzed using Power SYBR® Green PCR Master Mix (Applied Biosystems) on an Applied Biosystems Step One Plus Real-Time PCR System using a two-stage cycle of 95°C for 10 min for 1 cycle and 95°C for 15 s and 60°C for 1 min repeated for 40 cycles, followed by a dissociation stage. Threshold cycle (Ct) values were determined by setting a constant threshold at 0.2. Analyses were done in triplicate for each sample; gene expression was normalized using house-keeping genes GAPDH and UBC. IFN scores were calculated as described previously (3). Primer sequences used are shown in Supplemental Table 4.

### Measurement of Cytokine Levels

Serum BAFF levels were measured using enzyme-linked immunosorbent assay (ELISA) method using Quantikine® Human BAFF Immunoassay (RnD Systems, Minneapolis, MN). Serum IFN levels were measured using LEGENDPlex^TM^ human anti-virus response panel (BioLegend). IL-10 and IL-6 cytokines levels in cell culture supernatants were measured using LEGENDPlex^TM^ (BioLegend). Cytokine concentrations were determined from dilution curves of cytokine standards provided in the kit. Samples were analyzed on a CytoFLEX flow cytometer (BC) and data were generated using LEGENDPlex^TM^ 7.0 software (BioLegend).

### Autoantigen Array and Anti-nuclear Antibody Test

IgG and IgM autoantibody profiling was performed using Autoantigen Microarray Super Panel at the Microarray Core Facility, University of Texas Southwestern Medical Center (Dallas, TX) (https://microarray.swmed.edu/products/category/protein-array/). Serum samples were treated with DNAse I as described (27), diluted 1:50, and incubated with autoantigen array. The antibodies binding to the antigens was detected with Cy3-labeled anti-IgG and Cy5-labeled anti-IgM. The array plates were scanned with GenePix® 4400A Microarray Scanner and images analyzed using GenePix 7.0 software. The averaged net fluorescent intensity (NFI) of each autoantigen for each sample between two microarray runs was normalized to internal controls (directly spotted IgM or IgG). Normalized signal intensity for each antigen was normalized for total serum IgM or IgG levels. The normalized signal intensities were calculated as the ratio of SLE patient serum signal over the averaged signal of the healthy controls. Significance analysis of microarrays (SAM) was used to identify significantly increased autoreactivities. SAM function was implemented from the Web Mev (mev.tm4.org) and open source standalone Mev packages (travis-ci.org/dfci-cccb/mev). The following settings were used: two class unpaired, number of permutations = 1000, Number of K-nearest neighbors = 10, q-val = <5%, fold change >5.

The anti-nuclear antibody (ANA) test was performed using HEp-2 antigen Kit (MBL Bion, Des Plaines, IL). Slides were incubated for 4 h with undiluted culture supernatants, washed, and incubated for 2 h with biotin-conjugated goat anti-human IgG, followed by 1 h incubation with streptavidin-FITC. Slides were washed and mounted in mounting medium. Images were captured on Inverted DMIRB Leica microscope with Meta-Morph software at 20x magnification.

### *TLR7* Genotyping

cDNA samples were genotyped using fluorescently-labeled TaqMan MGB probes and analyzed on ABI 7900 HT real-time PCR system (Applied Biosystems). DNA of known genotype was used to validate the assays and as references in each experiment. *TLR7 rs3853839* risk (G) and non-risk (C) were defined based on Shen et al. (22). For the studies reported here, *TLR7 rs3853839* GG, CG or G genotypes were identified as risk allele carriers, and *TLR7 rs3853839* CC or C, as non-risk allele carriers.

### Statistical Analysis

Statistical tests were performed using the Prism 5.0 (GraphPad, San Diego). Non-parametric Mann-Whitney test was used to compare group values. Categorical variables were compared using Fisher's exact test. One-way ANOVA, Kruskal–Wallis test with Dunn's multiple comparison test was used to assess the significance of differences between groups. Two-way repeated measures ANOVA were used in some cases. Correlations were performed using non-parametric Spearman, two-tailed, rank-order method. Results are reported as mean ± SD; *p* ≤ 0.05 were considered statistically significant.

## Results

### High *TLR7* Expression in SLE Patient PBMCs

**Figure 1:**
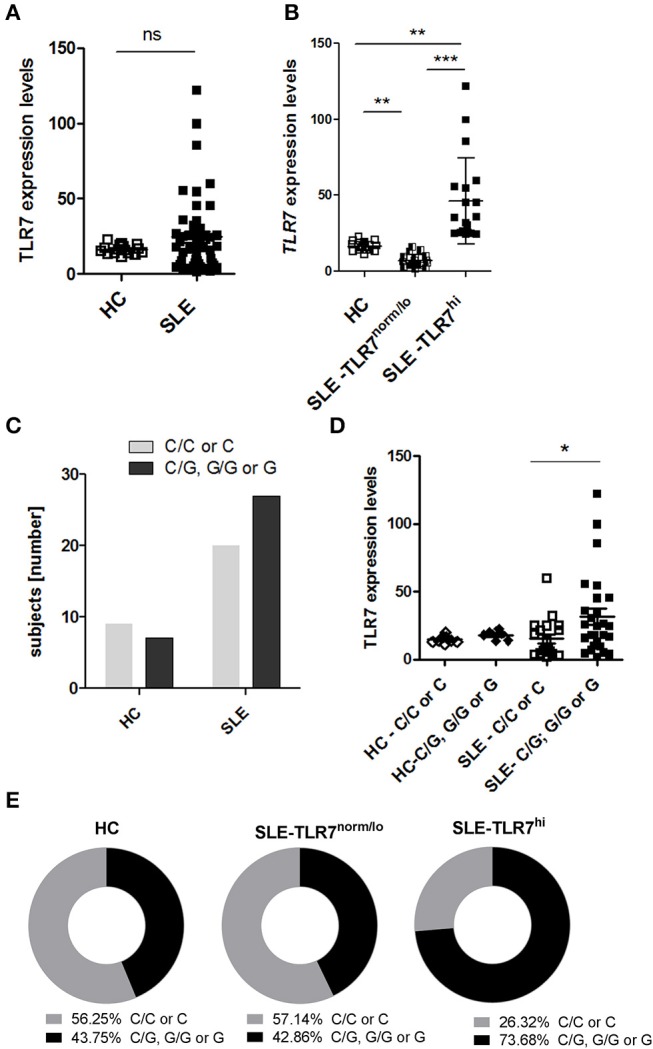
High *TLR7* expression in a subset of SLE patients. Peripheral blood mononuclear cells (PBMCs) were collected from healthy controls (HC) and SLE patients. The expression levels of *TLR7* mRNA were measured by RT-PCR. PCR data were normalized to the expression of housekeeping genes and is presented here as 2^Δ*CT*^x10^3^. **(A)** *TLR7* expression in PBMCs from HCs (*n* = 16) and SLE patients (*n* = 47) **(B)** SLE patients were divided into TLR7^norm/lo^ (*n* = 21) and TLR7^hi^ group (*n* = 19) based on their *TLR7* expression (TLR7^norm/lo^ SLE group: *TLR7* levels ≤ HC mean (2^Δ*CT*^x10^3^ ≤ 16.25) and TLR7^hi^ SLE group: *TLR7* > 2x SD above the HC mean [2^Δ*CT*^x10^3^ > 22.50)]. Each symbol represents an individual; horizontal lines indicate mean ± SD. *p < 0.05, **p < 0.01, and ***p < 0.001, significance determined by Mann-Whitney test or Kruskal-Wallis, with Dunn's post-test. **(C)** Genetic analysis of *TLR7 rs3853839* C and G allele frequencies. Graph shows the number of *TLR7 rs3853839 CC* or *C* (non-risk allele carriers) and TLR7 rs3853839 *GG, CG* or *G* (G-risk allele carriers) subjects in HC (*n* = 16) and SLE (*n* = 47) group (*P*-value = 0.39 by Fisher's exact test). **(D)** *TLR7* mRNA expression in HC (*n* = 16) and SLE subjects (*n* = 47), divided based on *TLR7 rs3853839 C/G* genotype. **(E)** Frequency of *TLR7 rs3853839 C/G* genotypes in HC (*n* = 16), TLR7^norm/lo^ (*n* = 21) and TLR7^hi^ (*n* = 19) SLE groups (*P*-value between TLR7^norm/lo^ and TLR7^hi^ SLE groups = 0.06 by Fisher's exact test).

To assess the effects of *TLR7* overexpression on peripheral B cell populations, we first analyzed the expression of *TLR7* in total PBMCs, isolated from 47 SLE patients and 16 healthy controls (HC). Real-time PCR (RT-PCR) analysis of *TLR7* mRNA expression showed variation in the expression levels of *TLR7* in the SLE group. Overall, we did not detect an increase in *TLR7* expression in SLE patients as compared to HC (Figure 1A). After setting a cut-off for the *TLR7* expression as the average mean of HC + 2SD, less than half (*n* = 19) of the SLE patients showed higher *TLR7* levels as compared to HC, which we designated here as the “SLE TLR7^hi^” group (Figure 1B). Twenty-one SLE patients had *TLR7* expression equal or lower to the HCs, which we designated the “SLE TLR7^norm/lo^” group (*n* = 21). 7 SLE patients were not included in either group, due to the cut-off. The TLR7^norm/lo^ and TLR7^hi^ SLE groups showed no statistical differences with respect to gender or age (Table 1). Notably, however, the overall disease activity was significantly higher in the TLR7^hi^ group, as compared to the SLE TLR7^norm/lo^ group (mean SLEDAI ± SD = 7.2 ± 3.37 vs. 3.80 ± 2.5; *p* = 0.0189). The number of patients with current renal involvement was also higher in the SLE TLR7^hi^ group (*p* = 0.0395, by Fischer's exact test).

The *TLR7 rs3853839 C/G* SNP is a functional SNP, associated with increased levels of *TLR7* mRNA and TLR7 protein expression (22, 24). To test whether the higher *TLR7* levels in the TLR7^hi^ SLE group was associated with the *TLR7 rs3853839 C/G* SNP, we separated SLE patients into two groups: non-risk allele carriers (C/C for females and C for males) and G-risk allele carriers (G/G or C/G for females and G for males). Genotypes of the HC and SLE groups are shown in Supplemental Table 2. Overall, the frequency of the *TLR7 rs3853839 G*-risk allele carriers was not significantly higher in SLE patients relative to HCs (*p* = 0.4, by Fisher's exact test) (Figure 1C). However, as previously reported (22, 24), *TLR7* mRNA levels were higher in G-risk allele carriers as compared to non-risk carriers SLE patients (Figure 1D). Next, we compared the frequency of the *TLR7* rs3853839 G-risk allele within the HC, TLR7^norm/lo^ and TLR7^hi^ SLE groups (Figure 1E). Results showed that 73.68 % of the TLR7^hi^ SLE patients were G-risk allele carriers, as compared to 42.86% in the TLR7^norm/lo^ SLE group and 43.75% in the HCs. The differences between SLE TLR7^hi^ and TLR7^norm/lo^ groups did not reach statistical significance (p-value = 0.06, by Fisher's exact test), but there was a clear trend toward G-risk genotype enrichment in the TLR7^hi^ group. Based on this data, we concluded that *TLR7 rs3853839 G*-risk allele is a strong factor in the *TLR7* increase but, since not all of the SLE TLR7^hi^ subjects had the *TLR7 rs3853839 G*-risk allele; other factors also are likely contributing to the increase of *TLR7* expression.

Consistent with the role of TLR7 in IFN production and previous reports which showed increases in ISG signature in *TLR7 rs3853839 G*-risk allele carriers (22, 24), the expression of ISGs (combined IFN scores) were higher in the SLE TLR7^hi^ group, as compared to TLR7^norm/lo^ SLE and HCs. Analysis of serum cytokine levels also showed increased levels of IFNα and IFNγ in the SLE-TLR7^hi^ group as compared to SLE-TLR7^norm/lo^ and HCs (Supplemental Figures 1A,B). The serum levels of IFNβ and IFNλ1/2 and other cytokines, including TNFα, IL-10, and IL-6 were not statistically different between the TLR7^norm/lo^ and TLR7^hi^ SLE groups (Supplemental Figures 1B,C).

### Changes in Peripheral B Cell Populations Associated With *TLR7* Overexpression

**Figure 2:**
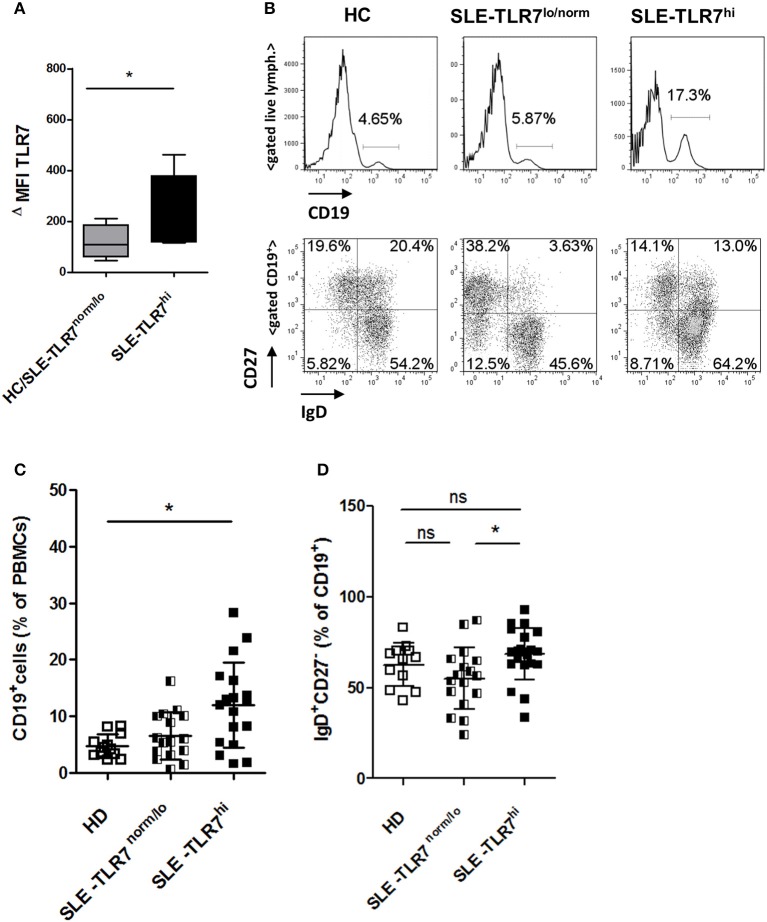
Changes in peripheral B cells associated with high *TLR7* expression **(A)** The expression of TLR7 in B cells analyzed by intracellular flow cytometry; graph shows normalized MFI of TLR7 protein expression in gated CD19^+^ B cells in HC/TLR7^norm/lo^ SLE group (*n* = 4) compared to TLR7^hi^ SLE group (*n* = 4); ΔMFI = MFI (anti-TLR7-PE signal)—MFI (Isotype-Ab-PE signal). Data are presented as mean ΔMFI ± SD, *p < 0.05 by Mann-Whitney test **(B)** Peripheral blood mononuclear cells (PBMC) from SLE patients and HCs were analyzed by flow cytometry. Representative flow cytometry plots of HC, TLR7^norm/lo^ SLE and TLR7^hi^ SLE groups, showing the frequency of CD19^+^ B cells of total PBMCs or gated CD19^+^ B cells stained with anti-CD27 and anti-IgD Abs, separating cells into IgD^+^CD27^−^ (naïve), IgD^−^CD27^+^ (switched memory), IgD^+^CD27^+^ (un-switched memory), and IgD^−^CD27^−^ (double-negative memory) subpopulations. Summary data of the frequencies of CD19^+^ cells in HC (*n* = 12), TLR7^norm/lo^ (*n* = 16) and TLR7^hi^ (*n* = 18) SLE groups **(C)** and the frequencies of IgD^+^CD27^−^ (naïve) B cells **(D)**. Differences between groups determined by Kruskal-Wallis, Dunn's post-test. Data are shown as the mean ± SD. *p < 0.05. (Additional data from the analysis of different B cell populations are presented in Supplemental Figure 2).

TLR7 expression in PBMCs is relatively restricted to pDCs and B lymphocytes, each of which comprises a minority population. We, therefore, asked if there was an intrinsic upregulation of *TLR7* expression in B cells. Analysis of TLR7 by intracellular (IC) flow analysis showed significantly higher TLR7 protein levels in CD19^+^ B cells from SLE TLR7^hi^ patients, as compared to TLR7^normal/lo^ or HC B cells (Figure 2A).

Next, we asked whether high *TLR7* was associated with any alterations in peripheral B cells. Flow cytometry analysis showed a significant increase in the percentage of CD19^+^ of total PBMCs in the TLR7^hi^ SLE group as compared to HCs (Figures 2B,C). Further analysis of the distribution of the B cell subsets using IgD and CD27 surface staining showed a slight, but statistically significant increase in naïve IgD^+^CD27^−^ B cells in the TLR7^hi^ SLE group as compared to TLR7^lo/norm^ SLE (Figures 2B,D). We also observed some differences in the frequencies of switched memory (IgD^−^CD27^+^) and double-negative (DN) memory B cells, but not un-switched memory (IgD^+^CD27^+^) B cells, between the two groups (Supplemental Figures 2A–D). The frequencies of CD19^+^CD11c^+^ B cells and CD38^++^CD27^++^ (plasma) cells were increased in SLE patients, but they were not statistically different between the TLR7^lo/norm^ and TLR7^hi^ SLE groups (Supplemental Figures 2E–G).

### Transitional B Cells Are Expanded in TLR7^hi^ SLE Patients

**Figure 3:**
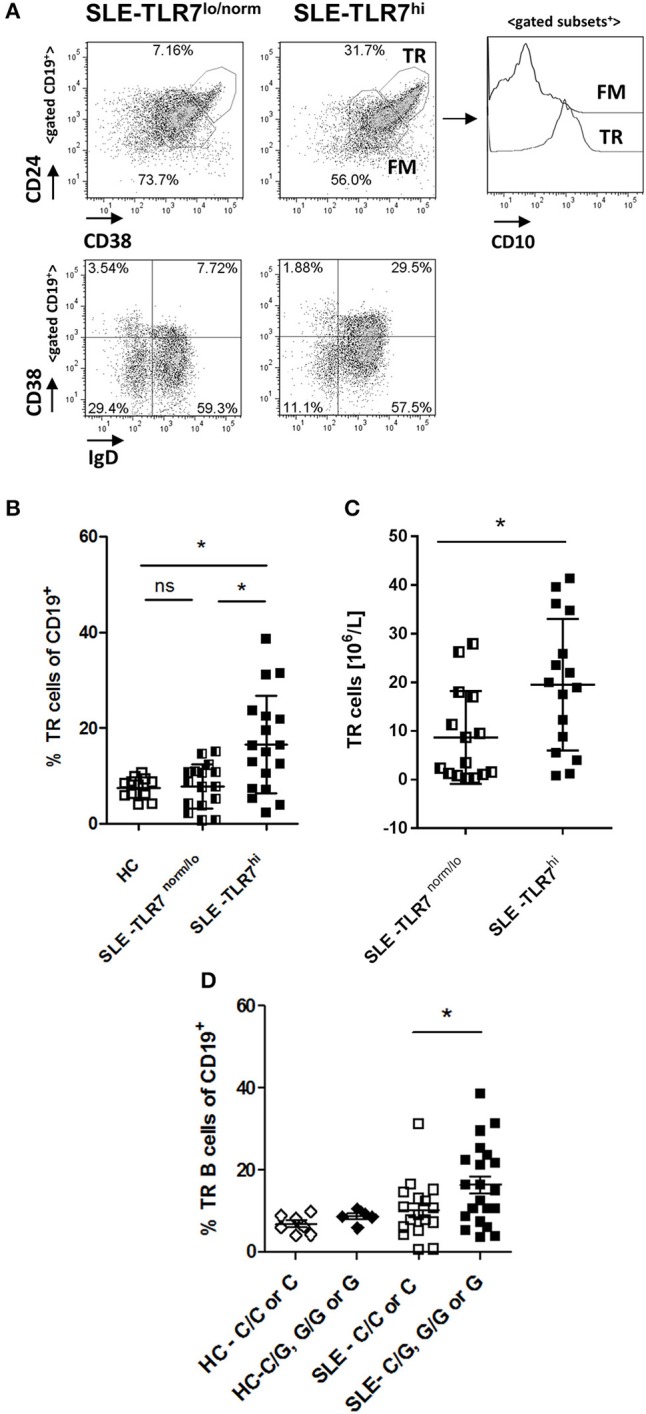
Transitional B cells are expanded in TLR7^hi^ SLE patients. **(A)** Representative flow plots of gated CD19^+^ cells from TLR7^norm/lo^ and TLR7^hi^ SLE patients. Transitional (TR) B cells defined as CD24^++^CD38^++^ or IgD^+^CD38^++^. Histograms to the right show CD10 expression in TR or follicular mature (FM) B cell subsets. **(B)** Summary data of the frequency of CD24^++^CD38^++^ CD10^+^ TR B cells in HC (*n* = 12), TLR7^norm/lo^ (*n* = 16), and TLR7^hi^ (*n* = 18) SLE patients. Differences between groups determined by Kruskal–Wallis, Dunn's post-test. Data are shown as the mean ± SD. *p < 0.05. **(C)** Bar graphs show absolute numbers of TR B cells in TLR7^norm/lo^ and TLR7^hi^ SLE groups calculated by multiplying available clinical lymphocyte counts for TLR7^norm/lo^ (*n* = 15) and TLR7^hi^ (*n* = 16) SLE patients by the fraction of TR B cells, determined by flow cytometry. Data are shown as the mean ± SD. *p < 0.05 by Mann-Whitney test. **(D)** TR B cells frequency in HC (*n* = 12) and SLE patients (*n* = 40) according to their *TLR7 rs3853839* C/G genotypes. Mann–Whitney test was used to compare non-risk (C/C or C) vs. risk (C/G, G/G, or G) allele carriers SLE patients *p < 0.05.

Mice overexpressing *Tlr7* have increased transitional (TR) B cells, and, when cultured *in vitro*, these TR B cells could produce autoantibodies (15). TR B cells are elevated in patients with juvenile dermatomyositis (JDM), particularly those with active disease (28). Therefore, we asked whether the increase of IgD^+^CD27^−^ naïve B cells in the TLR7^hi^ SLE group was due to increased TR B cells. Analysis of CD19^+^ B cells showed a higher percentage of CD38^++^CD24^++^CD10^+^ (TR) B cells in the TLR7^hi^ SLE patient group, as compared to the HC and TLR7^norm/lo^ SLE group (*p* = 0.0085, 1-way ANOVA) (Figures 3A,B). The expansion of TR B cells was also evident when TR B cells were identified as CD19^+^ IgD^+^CD38^++^ cells (Figure 3A and Supplemental Figure 3). The absolute number of TR B cells was also increased in the TLR7^hi^ SLE group, as compared to the TLR7^norm/lo^ SLE group (Figure 3C).

When SLE patients were stratified by their *TLR7 rs3853839 C/G* genotype, TR B cells were expanded in risk-allele carriers SLE patients, as compared to non-risk allele carriers (*p* = 0.05, Mann-Whitney two-tailed test) (Figure 3D). While the overall TR B cell frequencies in SLE patients correlated positively with *TLR7* expression (*R* = 0.339, *p* = 0.0088); we found no correlation between TR B cell frequencies and the expression of other TLRs, including *TLR9* (Supplemental Figures 4A,B), suggesting the effect on TR B cells is specific to TLR7. Since the B cell pro-survival cytokine BAFF regulates TR B cell survival and can be produced in response to type I IFNs, we also measured BAFF levels and compared it between the TLR7^norm/lo^ and TLR7^hi^ SLE groups. We found no significant differences in BAFF levels between the two groups and, no association between BAFF titers and *TLR7* expression and/or TR B cell expansion and BAFF titers (Supplemental Figures 4C,D).

Together, these results show that high *TLR7* expression is associated with changes in peripheral B cells, particularly, an increase in the percentage and the absolute number of TR B cells. The increase of TR B cells correlated with *TLR7* expression, but, was not dependent on *TLR9* expression and/or BAFF levels.

### Overexpression of *TLR7* Is Associated With Enrichment for More Autoantibody Specificities

**Figure 4:**
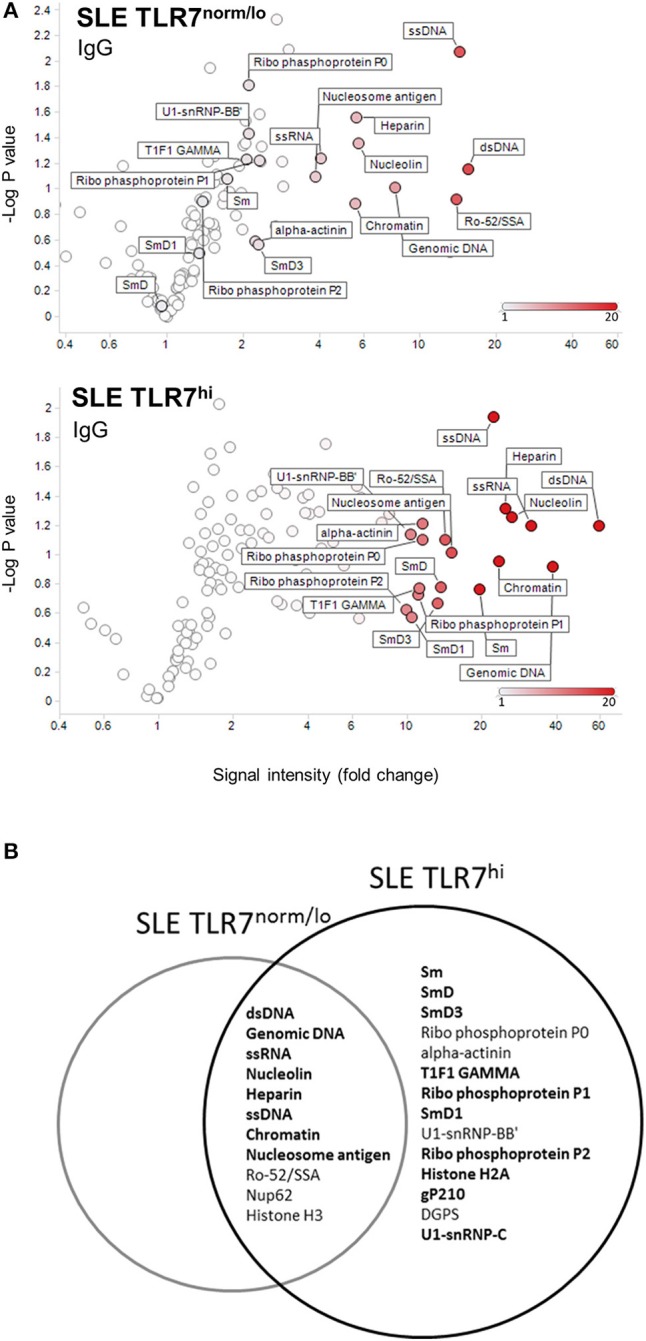
TLR7^hi^ SLE patients show increased IgG auto-Ab production and a broader range of autoreactive specificities. **(A)** Analysis of IgG antibody specificity in sera from SLE TLR7^norm/lo^ and SLE TLR7^hi^ groups by autoantigen microarray. Signal intensity for each antigen was normalized for total IgG levels. The normalized signal intensities were calculated as the ratio of SLE patient serum signal over averaged healthy control signal. Volcano plots depict averaged fold increase of normalized signal intensity of TLR7^norm/low^ (*n* = 8) and TLR7^hi^ (*n* = 8) SLE groups over averaged HC signal (*n* = 4). X-axis: fold increase in signal intensity of SLE groups *vs*. HCs. Y-axis: negative log10 of *p*-values obtained from two-tailed non-parametric *t*-tests comparing SLE patient and HCs serum autoantibody signal. Show are autoantigens with a signal >10-fold over HC in TLR7^low^ SLE patients (top) compared to TLR7^hi^ SLE patients (bottom). **(B)** Venn diagram shows top-hits autoantigens for each group, based on the Significance Analysis of Microarrays (SAM). Show are specificities with >5-fold change over the normalized signal intensity of HCs and SAM *q*-values <5%. Bold fonts designate a stronger signal in the SLE TLR7^hi^ group as compared to the TLR7^norm/lo^ SLE group. (SAM data is presented in Table 2).

To further understand how the increase in *TLR7* expression might affect B cells and auto-Ab production in SLE patients, we tested IgM and IgG autoantibodies in serum samples from HCs (*n* = 4), SLE TLR7^norm/lo^ (*n* = 8) and SLE TLR7^hi^ (*n* = 8) patients using autoantigen array. The IgM array showed little autoreactivity in the SLE samples; what little was detected was primarily directed against genomic DNA and chromatin with no notable differences between the two groups (data not shown). IgG array analysis revealed significant differences between the TLR7^norm/lo^ and TLR7^hi^ SLE groups. The signal intensity (increase over HCs) for multiple nuclear and non-nuclear auto-Ags was significantly higher in the TLR7^hi^ SLE group, as compared to TLR7^norm/lo^ SLE (Figure 4A). To better quantify differences between groups, we performed a significance analysis of microarrays (SAM). Using 5-fold increase over the normalized signal intensity of the HCs as a threshold, the TLR7^norm/lo^ group showed a significant (q value of less than 5%) increase in reactivity against eleven different auto-Ags; the TLR7^hi^ SLE group showed a significant increase in reactivity against twenty-five different auto-antigens. Fourteen different specificities, including Sm, SmD1, SmD3, U1-snRNP-BB”, U1-snRNP-C, Ribo-phosphoprotein-P1 (RPP1) and Ribo-phosphoprotein-P2 (RPP2) were increased in the TLR7^hi^ SLE group only (Figure 4B and Table 2). Reactivity against nineteen auto-Ags, including dsDNA, ssRNA, Nucleolin, ssDNA, Chromatin, Sm- and SmD-Ags, RPPs, U1-snRNP-C, Histone H2A, and gp210 were higher in the TLR7^hi^ SLE group, as compared to the TLR7^norm/lo^ group (Figure 4B and Table 2).

**Table 2:** Significance Analysis of Microarrays (SAM), data summary.

Autoantigens^*^	**SLE TLR7**^****norm/lo****^ **vs. HC**		**SLE TLR7**^****hi****^ **vs. HC**		**SLE TLR7**^****hi****^ **vs.** **SLE TLR7**^****norm/lo****^	
	**Fold change**	*q*-val^**^ (%)	**Fold change**	***q*-val** **(%)**	**Fold change**	***q*-val** **(%)**
dsDNA	15.42	0	52.85	0	3.43	0
Genomic DNA	7.94	0	34.32	0	4.32	0
ssRNA	4.05	0	28.71	0	7.09	0
Nucleolin	5.72	0	23.91	0	4.18	0
Heparin	5.6	0	22.41	0	4.01	0
ssDNA	14.28	0	21.16	0	1.48	3.5
Chromatin	5.57	0	21.04	0	3.78	0
Nucleosome antigen	3.85	0	13.63	0	3.54	0
Ro-52/SSA	13.94	0	12.83	0	5.40	ns
Nup62	2.71	0	5.73	0	2.11	ns
Histone H3	3.03	0	5.67	0	1.87	ns
Sm	1.74	ns	17.7	0	10.17	0
SmD	0.96	ns	12.36	0	12.93	0
SmD3	2.3	ns	11.93	0	5.18	4.99
Ribo phosphoprotein P0	2.11	ns	10.5	0	4.99	ns
Alpha-actinin	2.23	ns	10.4	0	4.66	ns
T1F1 GAMMA	2.06	ns	10.14	0	4.92	4.99
Ribo phosphoprotein P1	2.33	ns	10	0	4.30	3.88
SmD1	1.35	ns	9.35	0	4.42	3.88
U1-snRNP-BB'	2.11	ns	9.31	0	4.42	ns
Ribo phosphoprotein P2	1.38	ns	8.97	0	6.49	3.88
Histone H2A	1.93	ns	7.7	0	3.98	3.88
GP210	2.32	ns	7.25	0	3.13	3.5
DGPS	2.88	ns	6.49	0	1.37	ns
U1-snRNP-C	2.05	ns	5.99	0	2.93	3.33

* *IgG top hits (>5 fold HD vs. SLE TLR7^hi^ threshold). Autoantigens are ordered based on average signal fold change (HD vs. SLE TLR7^hi^)*.

** *q-factor determination of False Discovery Rate (FDR); q values <5% considered significant*.

*The following settings were used: two class unpaired, number of permutations = 1000, Number of K-nearest neighbors = 10, q-val = <5%, fold change >5. Hierarchical clustering was performed by the average linkage method calculated using the Mev package above*.

These results show that high *TLR7* is associated with an increase in auto-Ab reactivity, broadening of the autoantigen recognition, and, the production of anti-RNA/RNP-specific auto-Abs.

### TLR7^hi^ SLE Patients Have More Autoreactive B Cells

**Figure 5:**
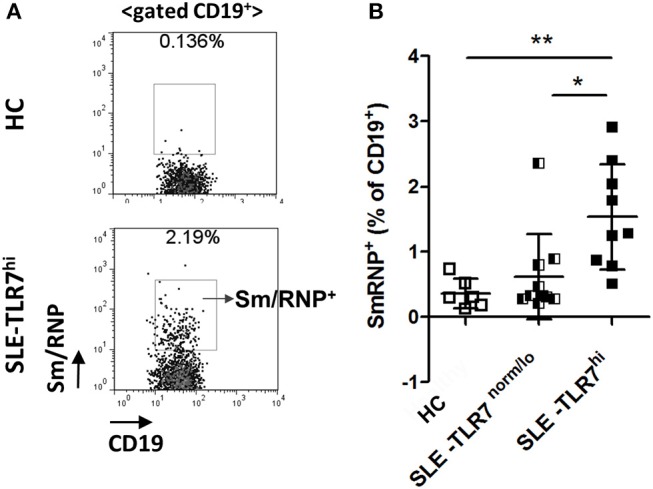
Analysis of Sm/RNP-specific B cells in TLR7^hi^ SLE patients. **(A)** Representative flow cytometry plots show the detection of CD19^+^ B cells reacting with Sm/RNP antigen in PBMC from HC and TLR7^hi^ SLE subjects. **(B)** Summary flow data of the frequencies of Sm/RNP-specific B cells of CD19^+^ in HC (*n* = 6), TLR7^norm/lo^ (*n* = 9), and TLR7^hi^ (*n* = 9) SLE patients. Differences between groups were determined by Kruskal–Wallis, Dunn's post-test. Data are shown as the mean ± SD, *p < 0.05 and **p < 0.01.

Consistent with the production of anti-Sm and RNP auto-Abs in TLR7^hi^ SLE patients, flow cytometry analysis using fluorescently labeled Sm/RNP showed a significant increase of Sm/RNP^+^ cells in the TLR7^hi^ SLE group, as compared to TLR7^norm/lo^ SLE and HCs (Figures 5A,B). The majority of Sm/RNP^+^ B cells were IgD^+^ (data not shown).

### Transitional B Cells Isolated From TLR7^hi^ SLE Patients Produce Autoantibodies *in vitro*

**Figure 6:**
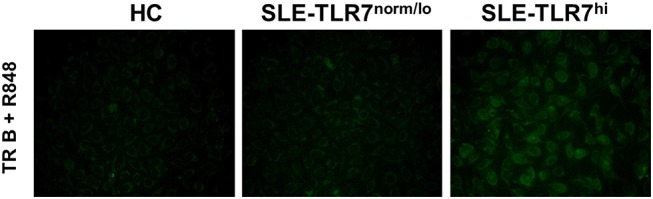
TLR7^hi^ TR B cells produce anti-nuclear auto-Abs. Representative HEp-2 ANA staining of undiluted culture supernatants from CD24^++^CD38^++^ TR B cells isolated from HC, TLR7^norm/lo^ and TLR7^hi^ SLE patients. Cells were cultured for 5 days in RPMI medium with TLR7 ligand-R848 (50 ng/ml). Data are representative of two independent experiments.

In mice overexpressing *Tlr7*, TR B cells are highly responsive to TLR7 ligation and, upon stimulation, produce auto-Abs *in vitro*. Since we found that TLR7^hi^ SLE patients have expanded TR B cells, we wanted to test whether TLR7^hi^ TR B cells could produce auto-Abs *in vitro*. We purified CD19^+^CD24^++^CD38^++^ TR from HCs, TLR7^norm/lo^ and TLR7^hi^ SLE patients. We obtained ~4,000 cells /donor and stimulated them with TLR7/8 ligand R848. After five days in culture, we collected cell culture supernatants and tested for autoreactivity using a HEp2 ANA staining assay. The results showed positive ANA staining in supernatants from TLR7^hi^ TR B cells, but not HC or TLR7^norm/lo^ TR B cells. TR B cells from two out of two TLR7^hi^ SLE patients showed a positive ANA signal (Figure 6). The signal was relatively weak, likely due to the low number of TR B cells in the culture. Nevertheless, these data show that, in response to TLR7 stimulation, TLR7^hi^ TR B cells are able to produce IgG auto-Abs. Due to the limited number of TR cells that can be obtained from a single donor we did not test TR B cell responses to other stimuli.

### Exposure of Healthy Transitional B Cells to IFNα Upregulates *TLR7* and Promotes Responses to TLR7-Ligation

**Figure 7:**
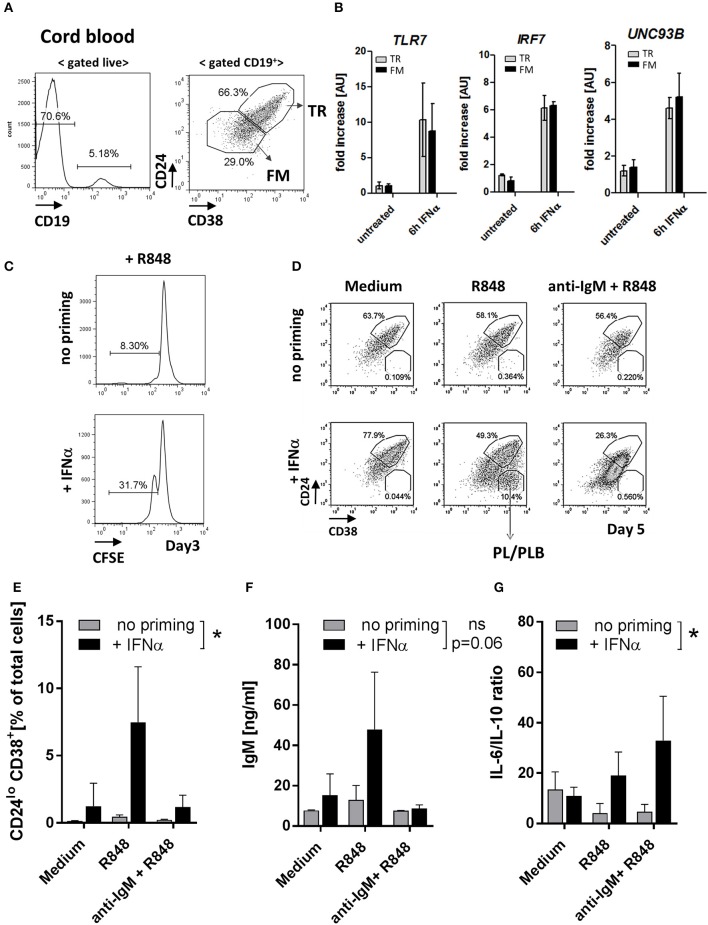
IFNα priming of transitional B cells promotes *TLR7* expression, cell differentiation and cytokine production in response to TLR7-ligand stimulation. Cord-blood (CB) transitional (TR) B cells were treated with IFNα and analyzed for the expression of *TLR7* and responsiveness to R848 stimulation *in vitro*. **(A)** Representative flow plots of B cell subsets in cord blood; shown is the gating of TR and follicular mature (FM) B cells based on CD24 and CD38 expression **(B)** TR or FM B cells were sort-purified and left untreated or stimulated with 1000 μ/ml IFNα for 6 h. The expression of *TLR7, IRF7*, and *UNC93B* in each subset was quantified by RT-PCR; data are presented as fold-change of mRNA levels relative to unstimulated cell (summary of three independent experiments). **(C)** Purified CB TR B cells were loaded with CFSE and left untreated or primed with IFNα and cultured for 3 days in RPMI medium. Representative histograms show the percentage of proliferating (CFSE^lo^) cells (data is representative of two independent experiments). **(D-G)** Purified CB TR B cells were primed with IFNα and stimulated with R848 (50 ng/ml) or a combination of R848 plus F(ab′)2 anti-human IgM (anti-IgM) (10 μg/ml) and cultured for 5 days. **(D)** Representative flow plots showing the percentages of TR B and plasma/plasmablast (PL/PLB) under different stimulatory conditions. **(E)** Summary data showing the percentage of CD24^−^CD38^++^ cells of live cells in cultures. **(F)** IgM titers in cell culture supernatants measured by ELISA. **(G)** Concentrations of IL-6 and IL-10 in cell culture supernatants measured by LEGENDPlex bead-based assay and presented as IL-6 to IL-10 ratios. Summary data of three independent experiments. No-priming and IFNα-priming conditions were compared using a 2-way repeated measure ANOVA.

To further test how TLR7 expression affects TR B cells, we used human umbilical cord blood (CB), which contains high frequencies of TR B cells that resemble peripheral blood TR B cells (29–31) Figure 7A and Supplementary Figure 5. *Ex situ*, CB-TR B cells expressed low levels of *TLR7*; however, exposure of CB-TR B cell to IFNα for 6 hours (“IFN-priming”) induced a significant (~ 5-10 fold) increase in *TLR7* expression and up-regulation of *UNC93B* and *IRF7*, other key molecules involved in TLR7 signaling (32, 33) (Figure 7B).

Consistent with the increase of TLR7 expression, IFNα priming enhanced CB TR B cell responses to TLR7 stimulation as measured by increased cell proliferation upon R848 stimulation (Figure 7C). We next sort-purified CB TR B cells, pre-treated them with IFNα for 6 hours or left them untreated, and stimulated them with either R848 alone or R848 in combination with F(ab′)2 anti-human IgM (anti-IgM). After five days in culture, we analyzed live cells by flow cytometry for the appearance and frequencies of CD24^++^CD38^++^ TR B, CD24^+^CD38^+^ follicular mature (FM) B cells, and CD24^−^/CD38^++^ plasma/plasmablast (PB/PBL) (Figure 7D). Samples that were not primed with IFN did not respond to R848 stimulation. However, when CB TR B cells were first primed with IFNα and then stimulated with R848, cells responded and differentiated into FM B cells or PB/PBLs (Figures 7D,E). Analysis of cell culture supernatants for Ig production showed an increase of IgM titers in the IFNα-primed/R848-stimulated samples, consistent with the appearance of PB/PBLs (Figure 7F). Under these conditions, CB-TR B cells did not produce significant amounts of IgG Abs (data not shown).

Since previous studies suggest a role for TR B cells in cytokine production (28, 34, 35), we also measured the production of IL-6 and IL-10 in the cell culture supernatants. IFNα-primed TR B cells showed high IL-6, but reduced IL-10 production in response to R848 and/or R848/anti-IgM stimulation, resulting in an increase in the IL-6/IL-10 ratios Supplemental Figure 6 and Figure 7G.

These data show that exposure of healthy TR B cells to IFNα induces a rapid increase in *TLR7* expression and promotes responses to TLR7 ligation, associated with cell differentiation, Ab-production and skewing toward a pro-inflammatory cytokine phenotype.

## Discussion

Previous studies strongly support the pathogenic role of TLR7 in murine lupus (4, 5, 8, 9, 14–18). SNPs in the *TLR7* gene have been previously associated with an increase in IFN production and SLE susceptibility (19, 22, 36), but, whether and how changes in *TLR7* expression may affect B cell activation and auto-Ab production in SLE is not well explored.

We found that TLR7^hi^ SLE patients showed significant expansion of the percentage and numbers of newly-formed transitional (TR) B cells. Autoantibody array assay showed increased auto-Ab reactivity in TLR7^hi^ SLE and enrichment for more autoantibody specificities, including the production of anti-RNA/RNP auto-Abs. Upon *in vivo* TLR7-ligand stimulation, TR B cells from TLR7^hi^ SLE patients produced IgG auto-Abs, suggesting that TR B cell might be an important source of pathogenic Abs in SLE.

Our *TLR7* gene expression data showed that SLE patients had variable levels of *TLR7;* however, a subset of SLE patients showed high *TLR7* expression as compared to HCs. This increase was associated with *TLR7 rs3853839 C/G* genetic variant, known to affect *TLR7* RNA turnover (22, 24). More than 70% of the TLR7^hi^ SLE patients had at least one G risk allele. This G-risk allele enrichment in the TLR7^hi^ SLE group may be due, in part, to the increased numbers of Asian SLE subjects in the TLR7^hi^ group, since the frequency of the G allele is considerably higher in Asians relative to Caucasians (0.777 *vs*. 0.168, respectively, data from 1000 Genomes). Since not all of the SLE TLR7^hi^ subjects carried the *TLR7 rs3853839 G*-risk allele, we propose that other factors also are likely to contribute to the increase of *TLR7* expression. Two other *TLR7* SNPs - *rs179019* and *rs179010* are associated with SLE susceptibility (20). Also, changes in miR-3148 can affect *TLR7* expression (24).

Increased TLRs expression in SLE PBMCs has been reported previously (37–39); TLR7 and TLR9, in particular, are more highly expressed in African-American SLE patients (39). These previous studies, however, did not examine if changes in *TLR7* expression might be linked to changes in peripheral B cells. More recently, Jenks et al. showed an increase in TLR7 signaling in DN2 (CXCR5^−^ CD21^−^ CD11c^+^ subset of IgD^−^CD27^−^ double-negative (DN)) memory B cells (40). The expansion of DN2 cells in African-American patients correlated with high disease activity, lupus nephritis and the presence of anti-Sm and anti-RNA autoantibodies. The increased TLR7 signaling in this study was not driven by high *TLR7* expression, but, linked low levels of the negative regulator TRAF5 (40).

Since TLR7 is predominantly expressed in pDCs and B cells and pDCs are present at <1% of PBMC in SLE (41), it is highly likely that our data reflect increased *TLR7* expression in B cells. This is supported by our results showing an increase in *TLR7* protein expression in CD19^+^ B cells. We found that high *TLR7* expression was associated with an overall increase in peripheral CD19^+^ B cells and a slight expansion of IgD^+^CD27^−^ naïve B cells. The frequencies of switched memory (IgD^−^CD27^+^) B cells were decreased in the TLR7^hi^, as compared to TLR7^norm/lo^ SLE group, whereas the frequencies of un-switched memory (IgD^+^ CD27^+^) B cells were comparable between the two groups. As previously described (42, 43), SLE patients in our cohort had increased (IgD^−^CD27^−^) double-negative (DN) memory B cells, but we found that frequencies DN B cells were lower in the TLR7^hi^, as compared to TLR7^lo/norm^ SLE group. These findings are somewhat surprising, given that increase of activated DN memory cells are found in SLE patients and the fact that DN memory B cells are highly responsive to stimulation through TLR9 and TLR7 (42–44). The overall expansion of DN memory B cell in our cohort was modest as compared to previous data that show significant accumulation of DN memory B cell in African-American SLE patients with active disease (32, 43). We found more Asians and no African-American patients in the TLR7^hi^ SLE group; however, African-American patients were underrepresented in our study. Thus, the discrepancy between our findings and previous studies might be due to differences in the demographics of the SLE patients. Other recent studies showed an association between SLE disease severity and increase in CD11c^+^ (Tbet^+^) cells (45, 46); but, similarly to DN cells, we did not find significant differences in CD11c^+^ B cell frequencies between the TLR7^lo/normal^ and TLR7^hi^ SLE groups.

Increases in circulating TR B cell is found in SLE and other autoimmune diseases, including Sjogren's Syndrome, Type 1 Diabetes and JDM (28, 47–49). Wu et al. reported that newly-diagnosed Asian SLE patients had an expansion of TR B cells, associated with increased PTEN expression (49). An increase in TR B cells, but a lack of DN B cell expansion was recently described in chronic cutaneous lupus (CCLE) patients (50). Also, Chang et al. showed a marked increase in the expression of p-SYK following IgM crosslinking of TR B cells from SLE patients (47). Another study has linked the expansion of TR B cells in the bone marrow of SLE patients to increases in IFNα levels and BAFF/APRIL production by neutrophils (51). Here, we did not detect a significant correlation between *TLR7* levels and BAFF expression. However, since neutrophils express low levels of *TLR7*, the increase in *TLR7* expression might have not directly affected their ability to secrete BAFF.

CD19^+^CD24^hi^CD38^hi^ TR B cells in healthy individuals have regulatory functions; in contrast, TR B cells from patients with SLE and RA fail to suppress T cell responses and have reduced ability to produce IL-10 (34). Piper et al. showed that TR B cells are expanded in patients with JDM and their expansion correlate positively with JMD disease activity (28). Protein and RNAseq analysis showed high interferon alpha (IFNα) and TLR7-pathway signatures in the TR B cells; furthermore, TLR7 and IFNα treatment of JDM B cells promoted IL-6 production *in vitro* (28). Another recent study reported an increase in TR B cells with a higher capacity to produce IL-6 in SLE patients (35). IFN-α treatment of TR B cell increased their survival by promoting NF-κB pathway and, by inhibiting the expression of the pro-apoptotic molecule Bax. The frequencies of TR B cell decreased in response to therapy (28, 35).

**Figure 8:**
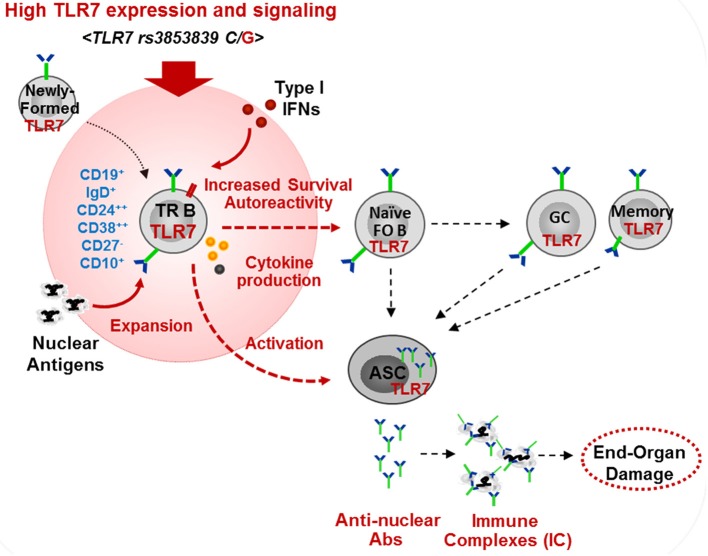
Model of the effects of high *TLR7* expression in driving transitional B cell expansion and auto-Ab production in SLE. High *TLR7* expression driven by *TLR7 rs3853839 (C/G)* polymorphism promotes the expansion of transitional (TR) B cells in the periphery. TLR7 signaling enhances TR B cell survival and, may directly promote TR B cells differentiation into Ab-secreting cells (ASC). TR and other B cells produce pathogenic anti-nuclear auto-Abs. TLR7-mediated type I IFN production induces *TLR7* expression and can further promote B cell activation.

We found that *TLR7* overexpression promoted the production of auto-Abs by TR B cells. This is consistent with our finding in *Tlr7*Tg mice, which showed the expansion and activation of TR B cells, capable of producing auto-Abs in response to TLR7-stimulation (15). Mouse TR B cells express AID and T-bet which can drive class-switching without the need for T-cell help (14, 15). Another recent study in lupus-prone BXD2 mice supports a link between TR B cell activation and *Tlr7* upregulation, driven by endogenous IFN-β production (52). Studies in human SLE also suggested that IFNβ production by TR B cells promotes cell survival and sensitivity to TLR7 and, is associated with more severe SLE disease manifestations in African-American patients (53). These findings, together with the data presented here, support the potential pathogenic role of TR B cells in SLE. Still, the exact contribution of TR B cell in the production of pathogenic auto-Abs needs further evaluation. TR B cells are highly enriched in poly/autoreactive specificities, suggesting they might be a potential source of auto-Abs (54). Post-GC B cells are considered a major source of class-switched auto-Abs, particularly anti-RNA/RNP Abs. However, Tipton et al. showed recently that a substantial fraction of Ab-secreting cell clones during SLE flares come from newly-activated naïve B cells (55). In light of this new finding, we suggest that auto-Ab producing cells may also directly arise from newly-activated TR B cells (Figure 8). We found that TLR7^hi^ TR B cells produce anti-nuclear auto-Abs in response to TLR7 ligation, suggesting that, upon auto-Ag encounter *in vivo*, they may produce auto-Abs. It is now well established that the transitional stage represents a stage in peripheral B cell development for reducing autoreactive specificities (56). Based on previous studies supporting a role for TLR7 and type I IFN signaling in B cell survival (35, 52, 57), we propose that high *TLR7* expression also promotes the survival of TR B cells, allowing more autoreactive B cells to reach the periphery, mature and become activated. In support of this model, we found more autoreactive B cells and a significant increase in auto-Ab production and a broader autoreactive B cell repertoire in TLR7^hi^ SLE patients. While our studies focused primarily on the effects of *TLR7* overexpression on TR B cells, one can predict that increased TLR7 signaling affects different stages of B cells development and promotes the activation of both naïve and memory B cells, and the formation of GCs, all of which can contribute to the production of pathogenic auto-Abs. Other factors, such as IFN signaling, BAFF, IL-21, IL-6, etc. can further cooperate with the TLR7 signal to drive B-cell activation.

*TLR7* mRNA expression correlates with anti-ENA positivity (58). Our in-depth autoantibody array profiling showed that TLR7^hi^ SLE patients have increased reactivity against nuclear Ags and, display a wider range of autoreactive specificities including, but not limited, to the production of anti-RNA/RNP auto-Abs. The number of SLE patients with current renal involvement was higher in SLE TLR7^hi^ group, which, might be due to increased anti-DNA and RNA/RNP Abs and, the formation of immune complexes. Two recent studies show an association between *TLR7 rs3853839 G* risk allele and the development of lupus nephritis or other manifestations of SLE, including malar rash, photosensitivity, pericardial effusion, reduced complement, and anti-dsDNA and anti-Sm auto-Abs (23, 59).

Our data on the expansion of TR B cells and their ability to produce auto-Abs *in vitro* support the B-cell intrinsic role of TLR7 signaling in the activation of TR B cells. However, *in vivo*, high TLR7 expression can also drive the activation of pDC and other myeloid cells and promote the production of type I IFNs (5, 10, 60). The presence of *TLR7 rs3853839* G-risk allele is associated with increased IFN production (22, 24). Consistent with the role of TLR7 in IFN production, we found high ISG scores and high IFNα levels in TLR7^hi^ SLE patients. Since TLR7 itself is an IFN-responsive gene and B cells express IFNAR, we cannot exclude that, *in vivo*, the increase of *TLR7* expression in B cells and changes in their phenotype are driven by type I IFNs. In addition to its effects on B cells, the TLR7-mediated type I IFN production is likely to affect other immune cells and to contribute to SLE disease manifestations (61).

TR B cells are present in very low numbers in the peripheral blood. To overcome this limitation, we used TR B cell isolated from umbilical cord blood (CB), which, phenotypically resemble TR B cells, found in the peripheral blood (29–31). Upon isolation, CB-TR B cells expressed low levels of TLR7 and did not respond to TLR7-ligand stimulation. These results are consistent with published data showing that human B cells responses to TLR7 ligation require exogenous IFNα (62). IFNα-priming of CB-TR B cells induced a robust up-regulation of TLR7 and other key molecules involved in TLR7 signaling, including IRF7 and UNC93B, also known to be upregulated in SLE patients (32, 33, 63). Thus, the IFN exposure of CB-TR B cells, at least to some extent, mimics *in vivo* inflammatory conditions. IFNα-priming promoted TR B cell responses to TLR7 ligand stimulation and also, altered their cytokine profiles. IFN priming alone was not sufficient to drive IgG and auto-Ab production by CB-TR B cells suggesting that, *in vivo*, other factors (*e.g.*, auto-Ag and different cytokines, such as BAFF or IL-21) might be required for the full activation of TR B cells. Our findings in CB-TR B cell cultures support that IFN-driven TLR7 upregulation promotes TR B cell activation and cytokine responses in response to stimulation; still, they might not precisely reflect what happens in an SLE patient.

Inflammatory conditions associated with an increase in IFNs production, such as viral infections, could promote TLR7 expression and drive Ab and cytokine production by TR B cells. Changes in *TLR7* expression can be driven by both genetic (*TLR7* SNPs), epigenetic (miRNAs and X- chromosome inactivation, XCI), and environmental factors, providing multiple links to the SLE pathology (22, 64, 65). Here, we showed that *TLR7 rs3853839 (C/G)* which affects *TLR7* expression and IFN production is associated with TR B cell expansion and increased auto-Abs production. It is of future interest to identify other genetic factors that might affect *TLR7* expression or its downstream signaling. It is likely that high *TLR7* expression, combined with other genetic factors may have additive or synergistic effects on SLE disease susceptibility. It is also possible, that different genetic factor, such as SNPs in the TLR7 or TRAF5 genes may affect different populations differently. The frequency of the *TLR7 rs3853839* G-risk allele is considerably higher in Asians, relative to Caucasians or African-Americans [C: 22%, G: 78% vs. C: 83%, G:17% and C:81%, G: 19% respectively (data from 1000 Genomes)], suggesting that the G-risk-associated phenotype might be more prevalent in Asian SLE patients. Changes in TLR7 signaling associated with IFNβ up-regulation or low TRAF5 expression, on the other hand, might be predominant in African-Americans SLE patients (40, 53). Thus, the association between TLR7 and B cell activation should be explored in larger and ethnically diverse SLE cohorts.

Our study shows a direct link between *TLR7* signaling and B cell abnormalities in SLE patients, specifically, the expansion of TR B cells and the increased production of auto-Abs. TR B cells in TLR7^hi^ SLE patients may represent a source of auto-Abs and pro-inflammatory cytokines. *TLR7* expression and *TLR7 rs3853839 (C/G*) genotyping can be useful biomarkers in SLE and other autoimmune diseases to help identify patients who might respond well to TLR7 and/or IFN blockade or B cell-targeted therapies.

## Data Availability

The raw data supporting the conclusions of this manuscript will be made available by the authors, without undue reservation, to any qualified researcher.

## Ethics Statement

All SLE subjects and healthy controls provided written informed consent. The study was approved by the University of Washington Institutional Review Board (IRB, STUDY00004154). Consent was obtained in accordance with the Declaration of Helsinki.

## Author Contributions

TW, JM, VT, and NG performed experiments and analyzed data. JC and KC performed genotyping and provided materials. Q-ZL performed protein array analysis. ML provided materials. TW, KE, XZ, and NG designed the study and wrote the manuscript.

### Conflict of Interest Statement

The authors declare that the research was conducted in the absence of any commercial or financial relationships that could be construed as a potential conflict of interest.

## Abbreviations

Ab: antibody
Auto-Ab: autoantibody
BAFF: B-Cell-Activating Factor
BCR: B-cell receptor
CB: cord blood
DN B cells: double–negative (memory) B cells
ISGs: interferon (IFN)-stimulated genes
IFN: interferon
PBMC: peripheral blood mononuclear cells
PCR: polymerase chain-reaction
RNP: ribonucleoprotein
SLE: systemic lupus erythematosus
TLR7: Toll-like receptor 7
TLR9: Toll-like receptor 9
TR B cells: Transitional B cells
ssRNA: single-stranded RNA
Sm: Smith Antigen.

## References

[1] Ahlin E Mathsson L Eloranta ML Jonsdottir T Gunnarsson I Ronnblom L. Autoantibodies associated with RNA are more enriched than anti-dsDNA antibodies in circulating immune complexes in SLE. Lupus. (2012) 21:586–95. 10.1177/0961203311434938 22300829

[2] Doedens JR Jones WD Hill K Mason MJ Gersuk VH Mease PJ. Blood-borne RNA correlates with disease activity and IFN-stimulated gene expression in systemic lupus erythematosus. J Immunol. (2016) 197:2854–63. 10.4049/jimmunol.1601142 27534558

[3] Hua J Kirou K Lee C Crow MK. Functional assay of type I interferon in systemic lupus erythematosus plasma and association with anti-RNA binding protein autoantibodies. Arthritis Rheum. (2006) 54:1906–16. 10.1002/art.21890 16736505

[4] Christensen SR Shupe J Nickerson K Kashgarian M Flavell RA Shlomchik MJ. Toll-like receptor 7 and TLR9 dictate autoantibody specificity and have opposing inflammatory and regulatory roles in a murine model of lupus. Immunity. (2006) 25:417–28. 10.1016/j.immuni.2006.07.013 16973389

[5] Deane JA Pisitkun P Barrett RS Feigenbaum L Town T Ward JM. Control of toll-like receptor 7 expression is essential to restrict autoimmunity and dendritic cell proliferation. Immunity. (2007) 27:801–10. 10.1016/j.immuni.2007.09.009 17997333 PMC2706502

[6] Diebold SS Kaisho T Hemmi H Akira S Reis e Sousa C. Innate antiviral responses by means of TLR7-mediated recognition of single-stranded RNA. Science. (2004) 303:1529–31. 10.1126/science.1093616 14976261

[7] Pisitkun P Deane JA Difilippantonio MJ Tarasenko T Satterthwaite AB Bolland S. Autoreactive B cell responses to RNA-related antigens due to TLR7 gene duplication. Science. (2006) 312:1669–72. 10.1126/science.1124978 16709748

[8] Santiago-Raber ML Dunand-Sauthier I Wu T Li QZ Uematsu S Akira S. Critical role of TLR7 in the acceleration of systemic lupus erythematosus in TLR9-deficient mice. J Autoimmun. (2010) 34:339–48. 10.1016/j.jaut.2009.11.001 19944565

[9] Eloranta ML Lovgren T Finke D Mathsson L Ronnelid J Kastner B. Regulation of the interferon-alpha production induced by RNA-containing immune complexes in plasmacytoid dendritic cells. Arthritis Rheum. (2009) 60:2418–27. 10.1002/art.24686 19644885

[10] Sakata K Nakayamada S Miyazaki Y Kubo S Ishii A Nakano K. Up-regulation of TLR7-mediated IFN-alpha production by plasmacytoid dendritic cells in patients with systemic lupus erythematosus. Front Immunol. (2018) 9:1957. 10.3389/fimmu.2018.01957 30210502 PMC6121190

[11] Kiefer K Oropallo MA Cancro MP Marshak-Rothstein A. Role of type I interferons in the activation of autoreactive B cells. Immunol Cell Biol. (2012) 90:498–504. 10.1038/icb.2012.10 22430248 PMC3701256

[12] Tsubata T. B-cell tolerance and autoimmunity. F1000Res. (2017) 6:391. 10.12688/f1000research.10583.1 28408984 PMC5373417

[13] Liu Z Bethunaickan R Huang W Lodhi U Solano I Madaio MP. Interferon-alpha accelerates murine systemic lupus erythematosus in a T cell-dependent manner. Arthritis Rheum. (2011) 63:219–29. 10.1002/art.30087 20954185 PMC3014995

[14] Berland R Fernandez L Kari E Han JH Lomakin I Akira S. Toll-like receptor 7-dependent loss of B cell tolerance in pathogenic autoantibody knockin mice. Immunity. (2006) 25:429–40. 10.1016/j.immuni.2006.07.014 16973388

[15] Giltiay NV Chappell CP Sun X Kolhatkar N Teal TH Wiedeman AE. Overexpression of TLR7 promotes cell-intrinsic expansion and autoantibody production by transitional T1 B cells. J Exp Med. (2013) 210:2773–89. 10.1084/jem.20122798 24145511 PMC3832927

[16] Hwang SH Lee H Yamamoto M Jones LA Dayalan J Hopkins R. B cell TLR7 expression drives anti-RNA autoantibody production and exacerbates disease in systemic lupus erythematosus-prone mice. J Immunol. (2012) 189:5786–96. 10.4049/jimmunol.1202195 23150717 PMC3544945

[17] Soni C Wong EB Domeier PP Khan TN Satoh T Akira S. B cell-intrinsic TLR7 signaling is essential for the development of spontaneous germinal centers. J Immunol. (2014) 193:4400–14. 10.4049/jimmunol.1401720 25252960 PMC4201954

[18] Walsh ER Pisitkun P Voynova E Deane JA Scott BL Caspi RR. Dual signaling by innate and adaptive immune receptors is required for TLR7-induced B-cell-mediated autoimmunity. Proc Natl Acad Sci USA. (2012) 109:16276–81. 10.1073/pnas.1209372109 22988104 PMC3479588

[19] Garcia-Ortiz H Velazquez-Cruz R Espinosa-Rosales F Jimenez-Morales S Baca V Orozco L. Association of TLR7 copy number variation with susceptibility to childhood-onset systemic lupus erythematosus in Mexican population. Ann Rheum Dis. (2010) 69:1861–5. 10.1136/ard.2009.124313 20525845

[20] Kawasaki A Furukawa H Kondo Y Ito S Hayashi T Kusaoi M. TLR7 single-nucleotide polymorphisms in the 3′ untranslated region and intron 2 independently contribute to systemic lupus erythematosus in Japanese women: a case-control association study. Arthritis Res Ther. (2011) 13:R41. 10.1186/ar3277 21396113 PMC3132023

[21] Lau CM Broughton C Tabor AS Akira S Flavell RA Mamula MJ. RNA-associated autoantigens activate B cells by combined B cell antigen receptor/Toll-like receptor 7 engagement. J Exp Med. (2005) 202:1171–7. 10.1084/jem.20050630 16260486 PMC2213226

[22] Shen N Fu Q Deng Y Qian X Zhao J Kaufman KM. Sex-specific association of X-linked Toll-like receptor 7 (TLR7) with male systemic lupus erythematosus. Proc Natl Acad Sci USA. (2010) 107:15838–43. 10.1073/pnas.1001337107 20733074 PMC2936646

[23] Wang CM Chang SW Wu YJ Lin JC Ho HH Chou TC. Genetic variations in Toll-like receptors (TLRs 3/7/8) are associated with systemic lupus erythematosus in a Taiwanese population. Sci Rep. (2014) 4:3792. 10.1038/srep03792 24445780 PMC3896912

[24] Deng Y Zhao J Sakurai D Kaufman KM Edberg JC Kimberly RP. MicroRNA-3148 modulates allelic expression of toll-like receptor 7 variant associated with systemic lupus erythematosus. PLoS Genet. (2013) 9:e1003336. 10.1371/journal.pgen.1003336 23468661 PMC3585142

[25] Gladman DD Ibanez D Urowitz MB. Systemic lupus erythematosus disease activity index 2000. J Rheumatol. (2002) 29:288–91. 11838846

[26] Tan EM Cohen AS Fries JF Masi AT McShane DJ Rothfield NF. The 1982 revised criteria for the classification of systemic lupus erythematosus. Arthritis Rheum. (1982) 25:1271–7. 10.1002/art.1780251101 7138600

[27] Li QZ Xie C Wu T Mackay M Aranow C Putterman C. Identification of autoantibody clusters that best predict lupus disease activity using glomerular proteome arrays. J Clin Invest. (2005) 115:3428–39. 10.1172/JCI23587 16322790 PMC1297234

[28] Piper C Wilkinson JMM Deakin GL Otto CT CD19(+)CD24(hi)CD38(hi) B Cells Are Expanded in juvenile dermatomyositis and Exhibit a Pro-Inflammatory Phenotype After Activation Through Toll-Like Receptor 7 and Interferon-alpha. Front Immunol. (2018) 9:1372 10.3389/fimmu.2018.01372 29988398 PMC6024011

[29] Palanichamy A Barnard J Zheng B Owen T Quach T Wei C. Novel human transitional B cell populations revealed by B cell depletion therapy. J Immunol. (2009) 182:5982–93. 10.4049/jimmunol.0801859 19414749 PMC2746373

[30] Guerrier T Youinou P Pers JO Jamin C. TLR9 drives the development of transitional B cells towards the marginal zone pathway and promotes autoimmunity. J Autoimmun. (2012) 39:173–9. 10.1016/j.jaut.2012.05.012 22695187

[31] Sims GP Ettinger R Shirota Y Yarboro CH Illei GG Lipsky PE. Identification and characterization of circulating human transitional B cells. Blood. (2005) 105:4390–8. 10.1182/blood-2004-11-4284 15701725 PMC1895038

[32] Kawai T Sato S Ishii KJ Coban C Hemmi H Yamamoto M. Interferon-alpha induction through Toll-like receptors involves a direct interaction of IRF7 with MyD88 and TRAF6. Nat Immunol. (2004) 5:1061–8. 10.1038/ni1118 15361868

[33] Kim YM Brinkmann MM Paquet ME Ploegh HL. UNC93B1 delivers nucleotide-sensing toll-like receptors to endolysosomes. Nature. (2008) 452:234–8. 10.1038/nature06726 18305481

[34] Blair PA Norena LY Flores-Borja F Rawlings DJ Isenberg DA Ehrenstein MR. CD19(+)CD24(hi)CD38(hi) B cells exhibit regulatory capacity in healthy individuals but are functionally impaired in systemic Lupus Erythematosus patients. Immunity. (2010) 32:129–40. 10.1016/j.immuni.2009.11.009 20079667

[35] Liu M Guo Q Wu C Sterlin D Goswami S Zhang Y. Type I interferons promote the survival and proinflammatory properties of transitional B cells in systemic lupus erythematosus patients. Cell Mol Immunol. (2019) 16:367–79. 10.1038/s41423-018-0010-6 29563616 PMC6461980

[36] Lee YH Lee HS Choi SJ Ji JD Song GG. Associations between TLR polymorphisms and systemic lupus erythematosus: a systematic review and meta-analysis. Clin Exp Rheumatol. (2012) 30:262–5. 22325161

[37] Klonowska-Szymczyk A Wolska A Robak T Cebula-Obrzut B Smolewski P Robak E. Expression of toll-like receptors 3, 7, and 9 in peripheral blood mononuclear cells from patients with systemic lupus erythematosus. Mediators Inflamm. (2014) 2014:381418. 10.1155/2014/381418 24692849 PMC3955595

[38] Komatsuda A Wakui H Iwamoto K Ozawa M Togashi M Masai R. Up-regulated expression of Toll-like receptors mRNAs in peripheral blood mononuclear cells from patients with systemic lupus erythematosus. Clin Exp Immunol. (2008) 152:482–7. 10.1111/j.1365-2249.2008.03646.x 18373699 PMC2453201

[39] Lyn-Cook BD Xie C Oates J Treadwell E Word B Hammons G. Increased expression of Toll-like receptors (TLRs) 7 and 9 and other cytokines in systemic lupus erythematosus (SLE) patients: ethnic differences and potential new targets for therapeutic drugs. Mol Immunol. (2014) 61:38–43. 10.1016/j.molimm.2014.05.001 24865418

[40] Jenks SA Cashman KS Zumaquero E Marigorta UM Patel AV Wang X. Distinct Effector B cells induced by unregulated toll-like Receptor 7 contribute to pathogenic responses in systemic lupus erythematosus. Immunity. (2018) 49:725–39 e6. 10.1016/j.immuni.2018.08.015 30314758 PMC6217820

[41] Murayama G Furusawa N Chiba A Yamaji K Tamura N Miyake S. Enhanced IFN-alpha production is associated with increased TLR7 retention in the lysosomes of palasmacytoid dendritic cells in systemic lupus erythematosus. Arthritis Res Ther. (2017) 19:234. 10.1186/s13075-017-1441-7 29052537 PMC5649081

[42] Jacobi M Reiter K Mackay M Aranow C Hiepe F Radbruch A. Activated memory B cell subsets correlate with disease activity in systemic lupus erythematosus: delineation by expression of CD27, IgD, and CD95. Arthritis Rheum. (2008) 58:1762–73. 10.1002/art.23498 18512812

[43] Wei C Anolik J Cappione A Zheng B Pugh-Bernard A Brooks J. A new population of cells lacking expression of CD27 represents a notable component of the B cell memory compartment in systemic lupus erythematosus. J Immunol. (2007) 178:6624–33. 10.4049/jimmunol.178.10.6624 17475894

[44] Giltiay NV Shu GL Shock A Clark EA. Targeting CD22 with the monoclonal antibody epratuzumab modulates human B-cell maturation and cytokine production in response to Toll-like receptor 7 (TLR7) and B-cell receptor (BCR) signaling. Arthritis Res Ther. (2017) 19:91. 10.1186/s13075-017-1284-2 28506291 PMC5433084

[45] Liu Y Zhou S Qian J Wang Y Yu X Dai D. T-bet(+)CD11c(+) B cells are critical for antichromatin immunoglobulin G production in the development of lupus. Arthritis Res Ther. (2017) 19:225. 10.1186/s13075-017-1438-2 28982388 PMC5629756

[46] Wang S Wang J Kumar V Karnell JL Naiman B Gross PS IL-21 drives expansion and plasma cell differentiation of autoreactive CD11c(hi)T-bet(+) B cells in SLE. Nat Commun. (2018) 9:1758 10.1038/s41467-018-03750-7 29717110 PMC5931508

[47] Chang NH Li TT Kim JJ Landolt-Marticorena C Fortin PR Gladman DD. Interferon-alpha induces altered transitional B cell signaling and function in systemic lupus erythematosus. J Autoimmun. (2015) 58:100–10. 10.1016/j.jaut.2015.01.009 25678471

[48] Habib T Funk A Rieck M Brahmandam A Dai X Panigrahi AK. Altered B cell homeostasis is associated with type I diabetes and carriers of the PTPN22 allelic variant. J Immunol. (2012) 188:487–96. 10.4049/jimmunol.1102176 22105996 PMC3670766

[49] Wu XN Ye YX Niu JW Li Y Li X You X. Defective PTEN regulation contributes to B cell hyperresponsiveness in systemic lupus erythematosus. Sci Transl Med. (2014) 6:246ra99. 10.1126/scitranslmed.3009131 25101889

[50] Wei HA Smith C Sanz K Lim I Drenkard C 2017 ACR/ARHP Annual meeting abstract supplement. Arthritis Rheumatol. (2017) 69(Suppl. 10):1–4426. 10.1002/art.40321 28960855

[51] Palanichamy A Bauer JW Yalavarthi S Meednu N Barnard J Owen T. Neutrophil-mediated IFN activation in the bone marrow alters B cell development in human and murine systemic lupus erythematosus. J Immunol. (2014) 192:906–18. 10.4049/jimmunol.1302112 24379124 PMC3907774

[52] Hamilton JA Wu Q Yang P Luo B Liu S Hong H. Cutting edge: endogenous IFN-beta regulates survival and development of transitional B cells. J Immunol. (2017) 199:2618–23. 10.4049/jimmunol.1700888 28904124 PMC5636672

[53] Hamilton JA Wu Q Yang P Luo B Liu S Li J. Cutting edge: intracellular IFN-beta and distinct type I IFN expression patterns in circulating systemic lupus erythematosus B cells. J Immunol. (2018) 201:2203–8. 10.4049/jimmunol.1800791 30201809 PMC6230322

[54] Meffre E Wardemann H. B-cell tolerance checkpoints in health and autoimmunity. Curr Opin Immunol. (2008) 20:632–8. 10.1016/j.coi.2008.09.001 18848883

[55] Tipton CM Fucile CF Darce J Chida A Ichikawa T Gregoretti I. Diversity, cellular origin and autoreactivity of antibody-secreting cell population expansions in acute systemic lupus erythematosus. Nat Immunol. (2015) 16:755–65. 10.1038/ni.3175 26006014 PMC4512288

[56] Chung JB Silverman M Monroe JG. Transitional B cells: step by step towards immune competence. Trends Immunol. (2003) 24:343–9. 10.1016/S1471-4906(03)00119-4 12810111

[57] Nundel K Green NM Shaffer AL Moody KL Busto P Eilat D. Cell-intrinsic expression of TLR9 in autoreactive B cells constrains BCR/TLR7-dependent responses. J Immunol. (2015) 194:2504–12. 10.4049/jimmunol.1402425 25681333 PMC4382804

[58] Chauhan SK Singh VV Rai R Rai M Rai G. Distinct autoantibody profiles in systemic lupus erythematosus patients are selectively associated with TLR7 and TLR9 upregulation. J Clin Immunol. (2013) 33:954–64. 10.1007/s10875-013-9887-0 23564191

[59] Raafat II El Guindy NR Shahin MH Samy LA El Refai RM. Toll-like receptor 7 gene single nucleotide polymorphisms and the risk for systemic lupus erythematosus: a case-control study. Z Rheumatol. (2018) 77:416–20. 10.1007/s00393-017-0283-7 28243744

[60] Buechler MB Teal TH Elkon KB Hamerman JA. Cutting edge: type I IFN drives emergency myelopoiesis and peripheral myeloid expansion during chronic TLR7 signaling. J Immunol. (2013) 190:886–91. 10.4049/jimmunol.1202739 23303674 PMC3552021

[61] Crow MK. Advances in understanding the role of type I interferons in systemic lupus erythematosus. Curr Opin Rheumatol. (2014) 26:467–74. 10.1097/BOR.0000000000000087 25010440 PMC4280994

[62] Bekeredjian-Ding IB Wagner M Hornung V Giese T Schnurr M Endres S. Plasmacytoid dendritic cells control TLR7 sensitivity of naive B cells via type I IFN. J Immunol. (2005) 174:4043–50. 10.4049/jimmunol.174.7.4043 15778362

[63] Nakano S Morimoto S Suzuki S Watanabe T Amano H Takasaki Y. Up-regulation of the endoplasmic reticulum transmembrane protein UNC93B in the B cells of patients with active systemic lupus erythematosus. Rheumatology. (2010) 49:876–81. 10.1093/rheumatology/keq001 20159909

[64] Souyris M Cenac C Azar P Daviaud D Canivet A Grunenwald S. TLR7 escapes X chromosome inactivation in immune cells. Sci Immunol. (2018) 3:eaap8855. 10.1126/sciimmunol.aap8855 29374079

[65] Miettinen M Sareneva T Julkunen I Matikainen S. IFNs activate toll-like receptor gene expression in viral infections. Genes Immun. (2001) 2:349–55. 10.1038/sj.gene.6363791 11607792

