# Supplementary material for: High TLR7 Expression Drives the Expansion of CD19+CD24hiCD38hi Transitional B Cells and Autoantibody Production in SLE Patients
Source: Front Immunol. 2019 Jun 4;10:1243. doi: 10.3389/fimmu.2019.01243 (PMC6559307; doi:10.3389/fimmu.2019.01243)
Supplement: Supplementary file 1 [file Data_Sheet_1.pdf]

Supplemental Table 1

Demographic characteristics of the healthy control group

Category	Feature	Healthy Controls (n=16)
Demographic	Age, mean ± SD	36.48 ± 8.32
	No. (%) female	12 (75.0)
	No. (%) male	4 (25.0)
	Race/Ethnicity, No (%)	
	Asian	6 (37.5)
	Back or African American	0 (0)
	Caucasian	8 (50.0)
	Hispanic	2 (12.5)
	Native Hawaiian/PI	0 (0)
	unknown/other	0

Supplemental Table 2

TLR7 expression and *TLR7 rs385839* genotype of SLE subject and healthy controls used in the study

study ID	gender	*TLR7* genotype	*TLR7* expression*
**SLE TLR7 ^{hi} group**			
SLE-1059	female	C/G	121.89
SLE-1077	male	G	99.69
SLE-1078	female	G/G	85.53
SLE-1074	male	C	59.68
SLE-870	female	G/G	55.49
SLE-941	female	G/G	54.61
SLE-985	female	C/G	45.27
SLE-1079	female	C/G	45.18
SLE-1075	female	G/G	35.82
SLE-1073	female	C/G	35.46
SLE-921	female	C/C	31.99
SLE-1020	female	C/G	30.33
SLE-1070	female	C/G	26.45
SLE-1004	female	G/G	25.50
SLE-1027	female	C/C	25.15
SLE-1069	female	C/C	24.94
SLE-1089	female	C/C	24.76
SLE-1076	female	C/G	24.36
SLE-984	female	C/G	24.30
**SLE TLR7 ^{int} group**			
SLE-922	female	C/C	21.30
SLE-840	female	C/C	18.73
SLE-1001	female	G/G	18.11
SLE-929	female	G/G	18.05
SLE-1090	male	G	17.78
SLE-828	female	C/C	17.33
SLE-1019	female	G/G	16.50
**SLE TLR7 $^{norm/lo}$ group**			
SLE-1098	female	C/G	15.82
SLE-1071	female	C/C	14.08
SLE-1086	female	G/G	12.79
SLE-824	female	G/G	10.03
SLE-1015	female	G/G	9.42
SLE-1097	female	C/C	8.81
SLE-845	female	C/C	8.57
SLE-1018	male	C	7.19
SLE-991	female	C/C	7.19
SLE-946	female	G/G	6.86
SLE-1085	female	C/C	6.28
SLE-1072	male	C	5.59
SLE-1034	female	G/G	5.09
SLE-1024	female	C/G	4.47
SLE-1933	female	C/C	4.49
SLE-1092	female	C/C	4.05
SLE-1023	male	G	3.79
SLE-1088	female	C/C	3.72
SLE-1055	female	C/C	2.86
SLE-872	female	C/C	1.91
SLE-1033	female	G/G	1.52

study ID	gender	*TLR7* genotype	*TLR7* expression*
**Healthy controls (HC)**			
HC1	female	G/G	17.28
HC2	female	C/G	13.70
HC3	female	C/G	20.22
HC4	female	C/G	16.57
HC5	female	C/C	14.07
HC6	female	C/C	15.89
HC7	female	C/G	15.22
HC8	female	C/C	22.62
HC9	female	C/C	13.17
HC10	female	C/C	13.80
HC11	male	C	13.00
HC12	male	G	19.10
HC13	female	C/C	19.85
HC14	female	C/G	18.00
HC15	male	C	11.15
HC16	male	C	16.40

* TLR7 expression levels are presented as $2^{-\Delta CT}$x10^3

Supplemental Table 3
Antibodies used for flow cytometry

Antibodies	Clone	Company
CD10	CB-CALLA	E-Bioscience
CD19	HIB19	Bio Legend
CD24	SN3	Invitrogen
CD27	LG.7F9	E-Bioscience
CD38	HB7	E-Bioscience
IgD	IA6-2	Bio Legend
TLR7	533707	R&D Systems
Mouse IgG2a isotype	MOPC-173	Bio Legend
Fc Block	Human TruStain FcX™	Bio Legend

Supplemental Table 4
Primer Sequences used for RT-PCR

Gene	Forward (5' to 3')	Reverse (3' to 5')
GAPDH	CAACGGATTTGGTCGTATT	GATGGCAACAATATCCACTT
IFI27	CTCTAGGCCACGGAATTAACC	CTCCTCCAATCACAACTGTAGC
IFI44L	GAACTGGACCCCATGAAGG	ACTCTCATTGCGGCACACC
IFIT1	CTCCTTGGGTTCGTCTACAAATTG	AGTCAGCAGCCAGTCTCAG
IRF7	TGGTCCTGGTGAAGCTGGA	GATGTCGTCATAGAGGCTGTTGG
MX1	AGCCACTGGACTGACGACTT	ACCACGGCTAACGGATAAG
PKR	CTTCCATCTGACTCAGGTTT	TGCTTCTGACGGTATGTATTA
TLR7	CATGCTCTGCTCTCTTCAACCAG	AGTGACATCACAGGGCAGAGTT
TLR9	GAAGGGACCTCGAGTGTGAA	CTGGAGCTCACAGGGTAGGA
UBC	GTCGCAGTTCTTGTTTGTGG	CAGCAAAGA TCAGCCTCTGC

Supplemental Figure 1

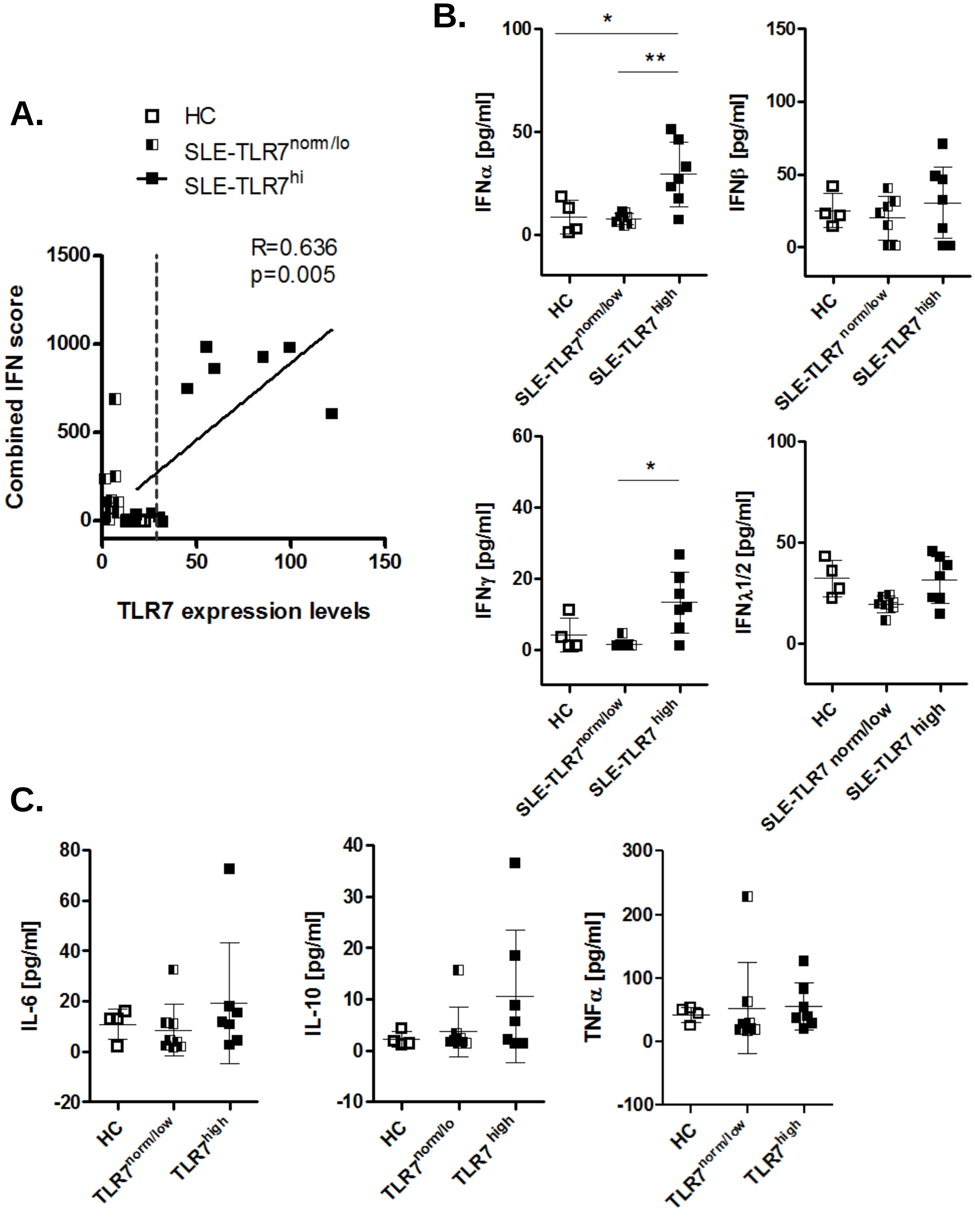

Supplemental Figure 1. Increased *TLR7* expression correlates with ISGs expression and IFNα and γ production. (A) Correlation between combined IFN scores and *TLR7* mRNA expression in PBMCs from HC (n=10) or SLE patients (TLR7$^{norm/lo}$ (n=10) and TLR7^{hi} (n=10)), defined by Spearman's correlation. IFN scores were calculated based on the relative expression of IFN-inducible genes (PKR, MX1, IFIT1, IFI27, and IFI44L). **(B and C)** Cytokine levels, measured in serum samples from HC (n=4), TLR7$^{norm/lo}$ (n=8) and TLR7^{hi} (n=7) SLE groups. Each symbol represents an individual, and horizontal lines indicate mean ± SD. *p<0.05 and **p<0.01, significance determined by Kruskal-Wallis, Dunn's post-test.

Supplemental Figure 2

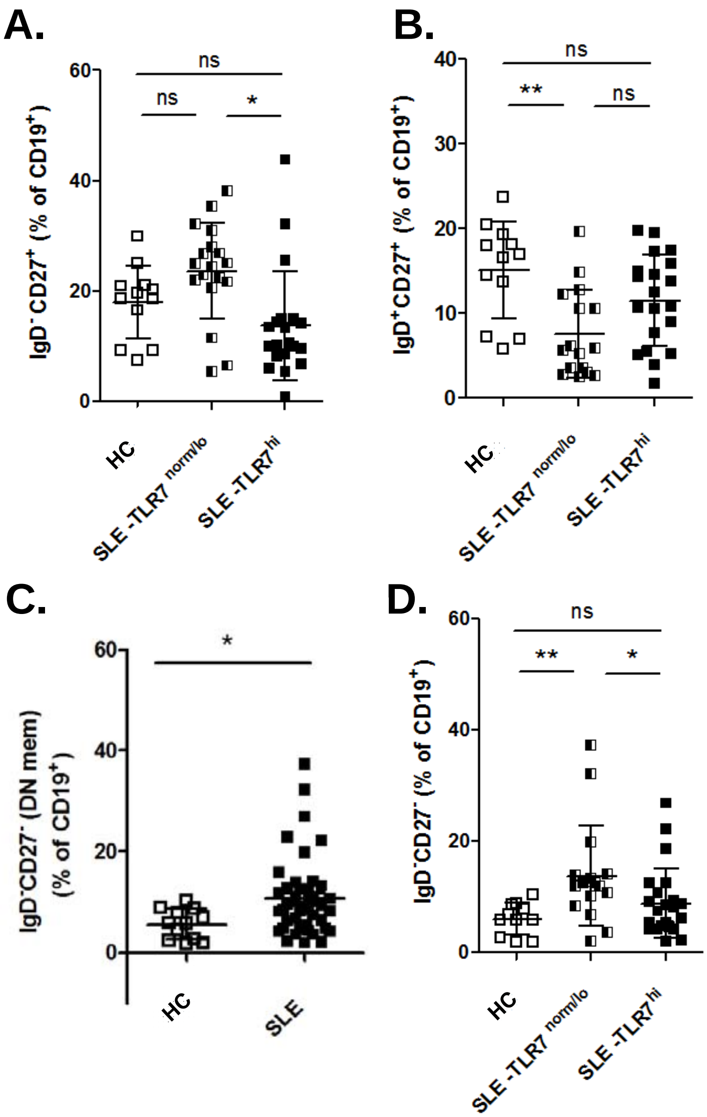

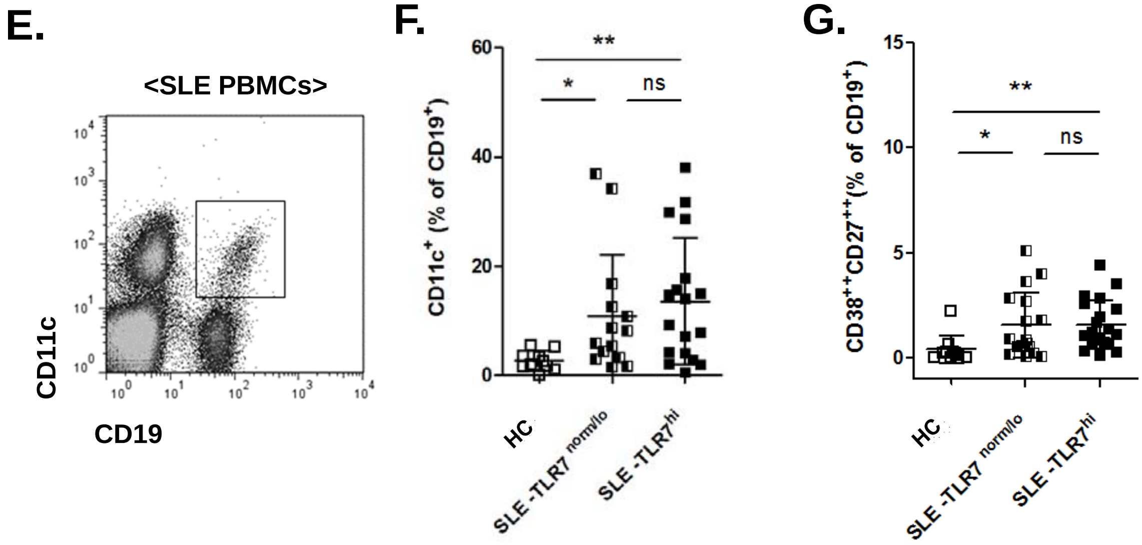

Supplemental Figure 2. Changes in peripheral B cells, associated with high *TLR7* expression in SLE patients. (A-B) Summary flow data of the frequencies of IgD^-CD27^+ (switched memory), IgD^+CD27^+ (un-switched memory) of $CD19^+$ cells in HC (n=12), $TLR7^{norm/lo}$ (n=16) and $TLR7^{hi}$ (n=18) SLE groups. **(C-D)** Summary flow data of the frequencies of IgD^-CD27^- double-negative (DN) memory B cells in HCs and SLE patients, or HC, $TLR7^{norm/lo}$ and $TLR7^{hi}$ SLE groups **(E-F)** Representative flow cytometry plots of SLE PBMCs showing staining of $CD19^+$ $CD11c^+$ B cells **(E)**. Summary flow data of the frequencies of $CD11c^+$ cells of $CD19^+$ cells in HC, $TLR7^{norm/lo}$ and $TLR7^{hi}$ SLE groups **(F)**. **(G)** Summary flow data of the frequencies of $CD38^{++}CD27^{++}$ plasma cells in HC, $TLR7^{norm/lo}$ and $TLR7^{hi}$ SLE groups. Differences between groups were determined by Mann-Whitney test (C) or Kruskal-Wallis, Dunn's post-test (A, B, D, F, and G). Data are shown as the mean ± SD. *$p<0.05$ and **$p<0.01$.

Supplemental Figure 3

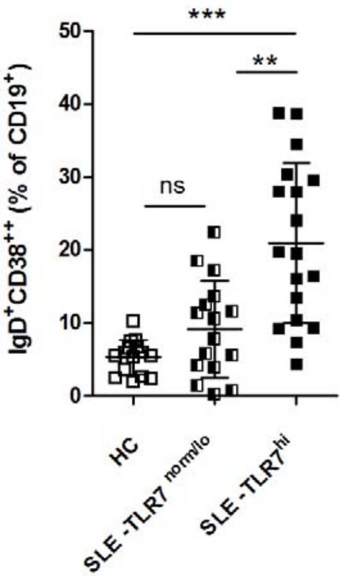

Supplemental Figure 3. Summary flow data of the frequency of TR B cells defined as IgD^+ $CD38^{++}$ of $CD19^+$ cells in HC (n=12), $TLR7^{norm/lo}$ (n=16) and $TLR7^{hi}$ (n=18) SLE groups. Data are shown as the mean ± SD. **p<0.01 and ***p<0.001 by Kruskal-Wallis, Dunn's post-test.

Supplemental Figure 4

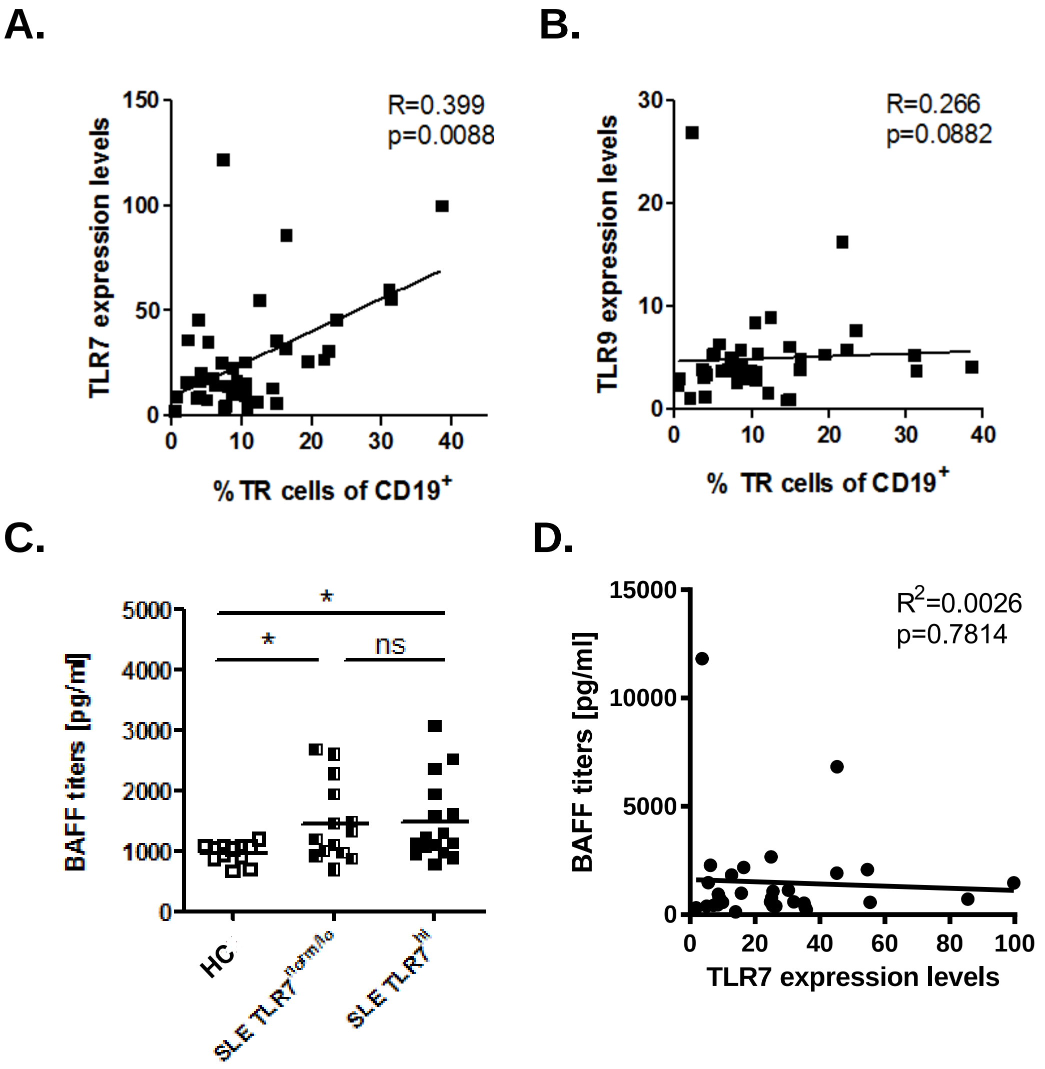

Supplemental Figure 4. (A and B) Correlations between TR B cell frequency and TLR7 or *TLR9* expression in SLE PBMCs (n=40). **(C)** BAFF levels in serum samples from HC (n=12), TLR7$^{norm/lo}$ (n=15) and TLR7^{hi} (n=17) SLE patients. **(D)** Correlation between BAFF serum titers and *TLR7* expression in SLE patients (n=32). Data are shown as the mean ± SD. *p<0.05 by Kruskal-Wallis, Dunn's post-test. Associations determined by Spearman's correlation.

Supplemental Figure 5

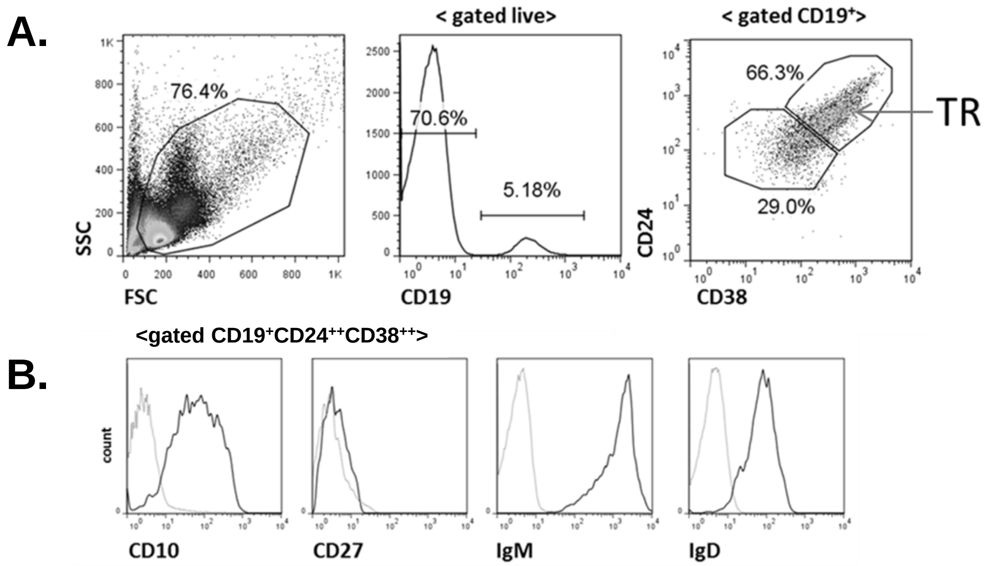

Supplemental Figure 5. Representative flow analysis of cord blood (CB) TR B cells. (A) Gating strategy used to identify CB-TR B cells using CD19, CD38, and CD24 staining. **(B)** CB-TR B cell phenotype. Histograms show staining with anti-CD10, anti-CD27, anti-IgM and anti-IgD Abs of gated CD19^{+}CD24^{++}CD38^{++} CB-TR B cells (black lines), or Ab-isotype controls (grey lines).

Supplemental Figure 6

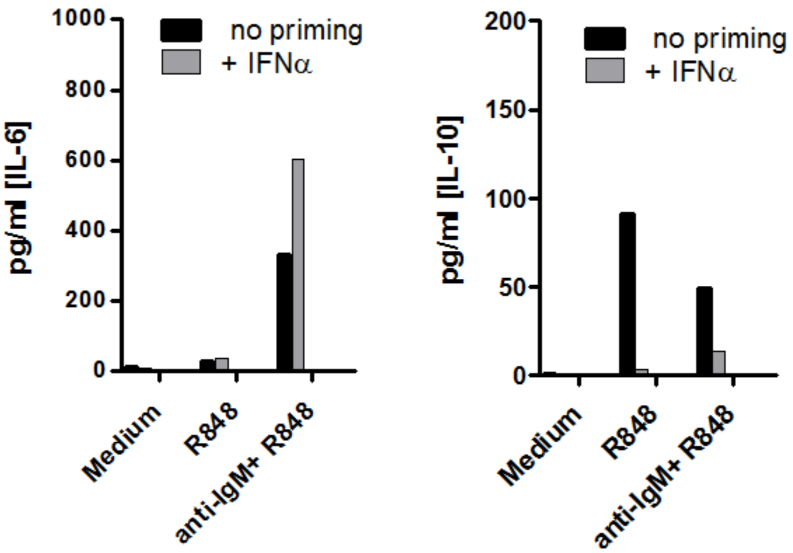

Supplemental Figure 6. IFN priming alters cytokine production by CB-TR B cells. Purified CB-TR B cells were primed with IFNα and cultured in cell medium, or, stimulated with R848 (50 ng/ml), or a combination of R848 plus F(ab′)2 anti-human IgM (anti-IgM) (10μg/ml) for 5 days. Concentrations of IL-6 and IL-10 were measured in cell culture supernatants. Representative data from one of three independent experiments.
